# Updating Prospective Self-Efficacy Beliefs About Cardiac Interoception in Anorexia Nervosa: An Experimental and Computational Study

**DOI:** 10.5334/cpsy.109

**Published:** 2024-06-26

**Authors:** Alkistis Saramandi, Laura Crucianelli, Athanasios Koukoutsakis, Veronica Nisticò, Liza Mavromara, Diana Goeta, Giovanni Boido, Fragiskos Gonidakis, Benedetta Demartini, Sara Bertelli, Orsola Gambini, Paul M. Jenkinson, Aikaterini Fotopoulou

**Affiliations:** 1 Department of Clinical, Educational and Health Psychology, University College London, UK; 2 Department of Biological and Experimental Psychology, Queen Mary University of London, London, UK; 3 Department of Health Sciences, University of Milan, Milan, Italy; 4 Aldo Ravelli Research Centre for Neurotechnology and Experimental Brain Therapeutics, University of Milan, Italy; 5 Department of Psychology, University of Milan-Bicocca, Milan, Italy; 6 Eating Disorders’ Unit, 1st Department of Psychiatry, National and Kapodistrian University of Athens, Greece; 7 Psychiatry Unit, ASST Santi Paolo e Carlo, S. Carlo General Hospital, Milan, Italy; 8 Psychiatry Unit, ASST Santi Paolo e Carlo, S. Paolo General Hospital, Milan, Italy; 9 Faculty of Psychology, Counselling and Psychotherapy, The Cairnmillar Institute, Melbourne, Australia

**Keywords:** Interoception, Anorexia Nervosa, Self-Efficacy, Belief Update, Bayesian Learning Framework, Metacognition

## Abstract

Patients with anorexia nervosa (AN) typically hold altered beliefs about their body that they struggle to update, including global, prospective beliefs about their ability to know and regulate their body and particularly their interoceptive states. While clinical questionnaire studies have provided ample evidence on the role of such beliefs in the onset, maintenance, and treatment of AN, psychophysical studies have typically focused on perceptual and ‘local’ beliefs. Across two experiments, we examined how women at the acute AN (N = 86) and post-acute AN state (N = 87), compared to matched healthy controls (N = 180) formed and updated their self-efficacy beliefs retrospectively (Experiment 1) and prospectively (Experiment 2) about their heartbeat counting abilities in an adapted heartbeat counting task. As preregistered, while AN patients did not differ from controls in interoceptive accuracy *per se*, they hold and maintain ‘pessimistic’ interoceptive, metacognitive self-efficacy beliefs after performance. Modelling using a simplified computational Bayesian learning framework showed that neither local evidence from performance, nor retrospective beliefs following that performance (that themselves were suboptimally updated) seem to be sufficient to counter and update pessimistic, self-efficacy beliefs in AN. AN patients showed lower learning rates than controls, revealing a tendency to base their posterior beliefs more on prior beliefs rather than prediction errors in both retrospective and prospective belief updating. Further explorations showed that while these differences in both explicit beliefs, and the latent mechanisms of belief updating, were not explained by general cognitive flexibility differences, they were explained by negative mood comorbidity, even after the acute stage of illness.

## Introduction

Interoception refers to the process of sensing, integrating, and interpreting internal, physiological signals (Khalsa et al., 2018). Interoceptive processing alterations have been proposed as a ‘transdiagnostic’ mechanism conferring vulnerability across mental health disorders (Nord et al., 2021), particularly those at the interface with physical health, such as eating disorders (EDs; Bruch, 1962; Jenkinson et al., 2018; Khalsa et al., 2022; Martin et al., 2019). For example, patients may present with an inability to sense a depleted nutritional body state and difficulties in perceiving and interpreting cutaneous, cardiac, and respiratory signals (e.g., Berner et al., 2018; Crucianelli et al., 2021; Davidovic et al., 2018; Kerr et al., 2016; Lapidus et al., 2020; Pollatos et al., 2008, 2016). Contemporary network analyses identify dysfunctional interoception as a core ED component (Brown et al., 2020; Cascino et al., 2019; Olatunji et al., 2018; Solmi et al., 2018; Vervaet et al., 2021), affecting treatment outcomes (Bizeul et al., 2001; Carter et al., 2004), unless targeted by interoceptive-based interventions (Baker, 2020; Choquette et al., 2023; Dalton et al., 2018; Heim et al., 2022; Khalsa et al., 2020).

Self-report has been mostly used to assess interoception in EDs, but recently psychophysical tasks have also revealed reduced interoceptive accuracy in clinical groups compared to controls (e.g., Bischoff-Grethe et al., 2018; Crucianelli et al., 2016, 2021; Garfinkel et al., 2015; Khalsa et al., 2015, 2018; Lutz et al., 2019; Pollatos et al., 2008, 2016). Interestingly, some studies have also shown group differences in the metacognitive evaluation and appraisal of interoceptive signals. Specifically, patients with Anorexia Nervosa (AN; characterised by severely restricted caloric intake and intense fears regarding weight gain; American Psychiatric Association, 2013) show lower confidence than controls in their interoceptive abilities, despite comparable performance levels (Khalsa et al., 2015; Kinnaird et al., 2020). These higher-order interoceptive processing levels have been termed interoceptive awareness, or metacognition (Critchley & Garfinkel, 2017; Garfinkel et al., 2015).

While however such psychophysical studies have focused on ‘local’ and ‘retrospective’ measures (e.g., trial-by-trial confidence-accuracy correspondence; Fleming & Lau, 2014; Garfinkel et al., 2015; Rouault, McWilliams et al., 2018), clinical traditions usually employ questionnaires (e.g., the Metacognitions Questionnaire; Cartwright-Hatton & Wells, 1997; Wells & Cartwright-Hatton, 2004) to sample explicit global, retrospective and prospective, metacognitive beliefs that have been found to be critical for the onset and maintenance of AN (for systematic reviews see: Palmieri et al., 2021; Sun et al., 2017). For example, metacognitive beliefs such as positive beliefs about worry and negative beliefs about thought uncontrollability and danger predict the drive for thinness in AN (Davenport et al., 2015; McDermott & Rushford, 2011; Palomba et al., 2017). Moreover, metacognitive dysfunctions in the form of ruminations over distorted cognitions pertaining to food, weight, and shape hinder the ability to engage in helpful cognitive processes such as problem solving (Safdari et al., 2013; Tchanturia et al., 2013). Patients may also show aberrant explicit beliefs about their illness and its causes (termed clinical insight; David, 2004). Insight deficits, common in restrictive AN (Greenfeld et al., 1991; Konstantakopoulos et al., 2011, 2012), indicate a specific metacognitive basis (Arbel et al., 2013). Additionally, beliefs about one’s capacity to succeed in, or cope with different situations and contexts (termed self-efficacy; Bandura, 1977) can be affected in EDs (Goodrick et al., 1999; O’Leary, 1985). More recently, network analysis studies suggest that metacognitive beliefs, such as ‘body mistrust’ may also determine the association between interoceptive ability and ED symptomatology (Olatunji et al., 2018; Monteleone & Cascino, 2021).

Yet, despite the frequent association of interoception and metacognition deficits with AN (Cooper et al., 2021; Jenkinson et al., 2018; Khalsa et al., 2022), theoretical insights from psychophysical paradigms have not been integrated with insights from clinical studies on explicit, clinically-relevant beliefs. Moreover, while the importance of global (mostly retrospective) metacognition in mental health is getting some recognition among experimental traditions (Seow et al., 2021), such insights have not been extended to prospective beliefs, or to interoception research in EDs (see Stephan et al., 2016 for a first theoretical proposal and model in relation to depression and self-efficacy). Bridging these gaps was the central aim of our interdisciplinary study.

Specifically, we used a unifying Bayesian, computational approach (Friston, 2010; Petzschner et al., 2017; Smith et al., 2020; Smith, Mayeli et al., 2021; Stephan et al., 2016) to study under one continuous framework how interoceptive perception and local metacognition influence the updating of explicit global, prospective beliefs about interoception. We have previously utilised this approach in different contexts to characterise the continuity between perceptual and metacognitive beliefs in self-awareness (Kirsch et al., 2021; Krahé et al., 2024). Here, we applied this unifying Bayesian approach to explicit, prospective and retrospective capability beliefs in the interoceptive domain. We assessed how information from one’s performance on a heartbeat counting task (HCT; Schandry, 1981) and related local and global retrospective beliefs about this performance (the level of accuracy-confidence correspondence) are combined to inform the updating of explicit, prospective metacognitive beliefs about one’s ability to monitor cardiac signals in AN. These investigations deepen our understanding of AN, shedding light on both the perception and evaluation of bodily signals in the here-and-now experience of the patient (“*How well did I feel my internal sensations*?”), but also on the processes that allow patients to use such ‘local’ perception and evaluation to update their ‘global’ interoceptive ability beliefs (“*How well do I perceive my internal sensations in general*?”).

Across two experiments we investigated how women at the acute AN stage, and post-acute AN phase (p-AN) and age-, ethnicity- and gender-matched healthy controls (HC) update their prospective (self-efficacy) beliefs about their heartbeat detection abilities after engaging in a modified HCT (Schandry, 1981). Comparisons across these three groups allowed us to disentangle state (e.g., changes present only during the acute AN phase as secondary neurocognitive, psychological, and physiological consequences to prolonged malnutrition) from trait mechanisms (premorbid deficits present in at-risk individuals, or deficits that endure beyond the acute phase, present during remission). While some theoretical (Barca & Pezzulo, 2020) and formal (Smith et al., 2020; Smith, Mayeli et al., 2021) approaches in EDs have used a similar Bayesian framework to characterise disruptions in interoceptive Bayesian inference in the perceptual domain (see Smith, Kirlic, et al., 2021 for a recent computational study showing precision-weighting differences between clinical groups, including a small (N = 14) ED sample, and healthy controls; and see Lavalley et al., 2023 for a recent replication), to our knowledge this approach has not been applied to explicit and prospective, or counterfactual metacognitive beliefs, typically identified as aberrant in EDs (see above). Thus, we developed a simplified interoceptive belief updating task and a corresponding Bayesian modelling approach to examine the key parameters involved in belief updating in the cardiac domain. When such metacognitive beliefs need to be updated, various sources of evidence, and corresponding precision and learning rate parameters are involved, and these include not only sensory signals and related beliefs but also cognitive beliefs about the underlying sensory beliefs and their precision (e.g., Kirsch et al., 2021; Krahé et al., 2024; Lavalley et al., 2023; Smith et al., 2020). Specifically, here we considered that the updating of explicit metacognitive beliefs can be influenced by at least two key sources of ‘evidence’: first, the perceptual performance itself (e.g., one’s actual accuracy) and second, one’s global retrospective beliefs about such performance (e.g., how accurate one thought they were after the task ends). Similarly, there can be different sources of ‘evidence precision’ and here we considered two experimental measures as ‘proxies’ for such precision – namely, individuals’ confidence about their performance during the task (which can be regarded as state-like beliefs about the accuracy of their interoceptive abilities) and individuals’ self-reported interoceptive abilities in everyday life as measured via a standardised questionnaire (which are more likely to be trait constructs, reflecting global beliefs about interoceptive abilities). This comparison of proxy measures of ‘evidence’ and of ‘precision of evidence’ allowed us to identify which combination best approximated the actual posterior beliefs of the participants. Crucially, we were able to explore if the clinical groups differ from controls in how much they take the ‘evidence’ into account (i.e., how much precision goes to the evidence vs. prior) when updating their prospective beliefs regarding cardiac interoceptive abilities. Moreover, although these two measures used as evidence precision proxies may appear as different at face value, they are the two measures we had of how people subjectively and retrospectively evaluate their interoceptive abilities retrospectively, either in everyday life (a trait-like measure of everyday, subjective evaluation of one’s interoceptive abilities), or in the lab. Thus, we were able to create alternative models of how our groups use ‘state-like’ or ‘global, trait-like’ retrospective beliefs about their interoceptive abilities when updating their self-efficacy beliefs about such abilities prospectively.

## Methods: Experiment 1

### Participants

The sample consisted of N_AN_ = 51, N_p-AN_ = 47, and N_HC_ = 63 women aged between 18 and 45 (full details on eligibility criteria, participant characteristics, and recruitment sites in Supplementary Material and Table S1). AN patients met the restrictive subtype AN DSM-5 criteria (American Psychiatric Association, 2013) and had a BMI < 18.5. Given growing concerns around weight-restoration criteria (e.g., Harrop et al., 2021; Khalsa et al., 2017; Lebow et al., 2018; Ralph et al., 2022), we chose a combination of objective and clinical criteria to best represent the patients’ clinical reality (see also Jenkinson et al., 2023). Therefore, instead of relying only on BMI criteria, which may inadequately reflect the clinical complexity of the AN recovery stages and symptom evolution, p-AN participants were eligible if they no longer met the DSM-5 criteria for restrictive subtype AN criteria according to their psychiatrist and met at least two of the following: BMI > 16.5, clinical and behavioural signs of AN recovery (e.g., no restrictive eating patterns) for at least 6 months, and/or a global Eating Disorders Examination Questionnaire (EDE-Q; Fairburn & Beglin, 1994) score <4. Additionally, if an p-AN participant had a BMI between 16.5 and 18.5 their clinical status was further confirmed by their experienced clinical team. HCs had a BMI between 18.5 and 25 and were excluded if they or a first-degree relative had an ED history.

### Design and Data Analysis

We used a revised version of the existing Heartbeat Counting Task (HCT; Schandry, 1981) to measure interoceptive belief updating. The task included the traditional measure of interoceptive accuracy, hereafter referred to as Performance, and three additional measures to examine participants’ beliefs about their performance before and after completing the HCT. These measures were participants’ (1) Prior Prospective Self-Efficacy Beliefs (i.e., how well they think they will do on the HCT), (2) Posterior Retrospective Self-Efficacy Beliefs (i.e., how well they think they performed on the task), and (3) Post-False Feedback Retrospective Self-Efficacy Beliefs (i.e., participants’ second retrospective evaluation of their Performance after receiving arbitrary feedback). In other words, after participants gave their Posterior Retrospective Self-Efficacy Belief, half of the participants were told they did much better than the others, while the other half were told they did much worse. Participants then rated their Performance retrospectively. These measures allowed to examine how prospective beliefs about HCT are generated prospectively and how they are updated retrospectively, after completing the HCT.

In a linear regression we first examined the effect of Group (independent variable; IV) on Prior Prospective Self-Efficacy Beliefs (dependent variable’ DV), expecting the AN group to be significantly more pessimistic about their heartbeat counting abilities than the HCs. Next, using a linear regression, we examined differences in Performance scores, expecting to not find significant group differences. To obtain a Performance percentage score we used the following Schandry (1981) transformation (1).


1 \[
Performance = \left[ {1 - \frac{1}{3} \sum \nolimits \frac{{\left| {Recorded\,\,Heartbeats\,\, -\,\, Counted\,\,Heartbeats} \right|}}{{Recorded\,\,Heartbeats}}} \right]*100
\]

Typically, Performance scores range from 0 (worst Performance) to 1 (best Performance), but here we multiplied the score by 100 to maintain consistency with our other measures, namely participants’ Prior Prospective and Posterior Retrospective Self-Efficacy Beliefs.

Then, in a linear regression we assessed whether the three Groups (IV) differed in their Posterior Retrospective Self-Efficacy Beliefs when controlling for the Prior Prospective Self-Efficacy Beliefs and Performance. We also calculated the difference between Performance and Prior Prospective Self-Efficacy Belief (i.e., Prediction Error) and examined how Prediction Error explained the AN group’s Posterior Retrospective Self-Efficacy Beliefs, and the between-group differences in Prediction Error. Finally, in within-group analyses we assessed whether false feedback influenced participants’ Post False-Feedback Retrospective Self-Efficacy Beliefs (after first controlling that positive false feedback was not randomly given only to participants with higher Performance scores and negative false feedback to participants with low Performance scores; see Supplementary Material, Table S4).

Exploratory regressions with psychometric traits and clinical characteristics (e.g., illness duration and severity) were run but these are presented in detail in the Supplementary Material (see Tables S5–S7) given that the purposes of *Experiment 1* (also preprinted and available here: https://psyarxiv.com/rntsf/; Saramandi et al., 2022) were to present our key results upon which we contextualised and based our preregistered *Experiment 2*.

### Main Experimental Measures and Procedure

Following baseline, demographic, and psychometric assessments (see below), participants wore the Polar heart rate monitor (model RS 800CX; see Emanuelsen et al., 2015; Fischer et al., 2016) on their left wrist and the heart rate monitoring throughout the experiment was explained to them. Next, participants silently sat on a chair with their legs uncrossed and their wrist gently resting on the table in front of them to obtain a 5-min baseline recording of their heart rate (used in *Experiment 1* as a control measure; see Supplementary Material, Tables S1, and S3).

Then, participants provided a Prior Prospective Self-Efficacy Belief estimate and proceeded to complete the HCT in the same, relaxed position, with their eyes open or closed (depending on what felt comfortable to them), as they were during the baseline heart rate measurement. Participants were asked to not attempt any physical manipulation to facilitate heartbeat detection and only report the number of heartbeats they actually felt rather than guess how many heartbeats they think they felt. Participants completed three heartbeat counting trials (25s, 45s, and 65s, with 30s rest breaks in between) in a randomised order between participants and information about the length of counting phases or participant Performance was not given. Participants were prompted with ‘*Go*’ and ‘*Stop’* signals at the start and end of each counting phase, respectively, and then verbally reported the number of felt heartbeats. After completing the HCT participants rated their Performance (Posterior Retrospective Self-Efficacy Belief). Finally, participants were given arbitrary false feedback regarding their Performance and were asked to provide a further estimate, namely the Post False-Feedback Retrospective Self-Efficacy Belief (due to clinical time constraints this measure is missing from N = 32 participants). Participants were fully debriefed at the end of the task and told that the feedback was for experimental purposes only, and not a reflection of their actual Performance during the task.

## Results: Experiment 1

The AN, but not p-AN group, significantly underestimated their performance abilities before completing the HCT as seen by their lower Prior Prospective Self-Efficacy Beliefs in comparison to those of the HCs (AN: *β*(SE) = –13.635(*4*.36), *t* = –3.23, *p* = .002, *R*^2^_ADJ_ = 0.06; p-AN: *β*(SE) = –4.12(*4*.50), *t* = –0.92, *p* = .362, *R*^2^_ADJ_ = 0.06). However, we found very small and non-significant differences between our clinical group’s Performance relative to the HCs (AN: *β*(SE) = 0.03(*.04*), *t* = .652, *p* = .516, *R*^2^_ADJ_ = –0.005; p-AN: *β*(SE) = 0.05(*.04*), *t* = 1.127, *p* = .262, *R*^2^_ADJ_ = –0.005) in line with recent findings (e.g., Kinnaird et al., 2020; Richard et al., 2019; but see Pollatos et al., 2008, 2016 for contrary findings). Moreover, AN, but not p-AN participants, had lower Posterior Retrospective Self-Efficacy Beliefs regarding their Performance compared to HCs (AN: *β*(SE) = –13.11(*3.75*), *t* = –3.497, *p* = .001, *R*^2^_ADJ_ = .48; p-AN: *β*(SE) = –1.46(*3.78*), *t* = –0.385, *p* = .701, *R*^2^_ADJ_ = 0.48). That is, AN participants thought they performed worse than HCs. Further analyses suggested this difference could be explained by the significant interaction between Group and Prediction Error, with the AN group showing a greater Prediction Error than HCs (i.e., AN patients predicted to perform worse than they actually did), and that difference in Prediction Error between the groups predicted their differences in Posterior Retrospective Self-Efficacy Beliefs (AN: *β*(SE) = –0.419(*0.17*), *t* = –2.538, *p* = .012, *R*^2^_ADJ_ = .159; p-AN: *β*(SE) = –0.03(*0.18*), *t* = –0.184, *p* = .855, *R*^2^_ADJ_ = 0.159).

When assessing the influence of external feedback in performance evaluation, positive false feedback about one’s performance resulted in higher Post False-Feedback Self-Efficacy Beliefs, and the opposite was observed following negative false-feedback within each group separately (AN: β(SE) = 37.49(*7.44*), *t* = 5.036, *p* < .001, *R*^2^_ADJ_ = .346; p-AN: β(*SE*) = 33.50(*5.96*), *t* = 5.62, *p* < .001, *R*^2^_ADJ_ = 0.44; HC: β(*SE*) = 24.72(*6.29*), *t* = 3.93, *p* < .001, *R*^2^_ADJ_ = –0.26). Such external, social feedback (even false, as in this case) may thus be used to modify self-efficacy beliefs. This finding further suggests that the belief updating difficulties we observed in the AN sample may be particularly compromised regarding interoceptive signals and not generalise to other domains, but future studies should examine if this applies to other perception domains, such as visual perception, before conclusions can be drawn for our findings’ domain-specificity.

### Summary of Experiment 1 results which led to Experiment 2 aims and design

Broadly, our preliminary findings suggest that AN patients have low self-efficacy about their cardiac, interoceptive abilities before they even engage with a task (prospectively), and seem to not be updating their self-efficacy beliefs retrospectively, despite not finding evidence of Performance group differences. Instead, they somehow adhere to their prospective, self-efficacy beliefs. Based on these results, we enhanced our task and preregistered the following experimental and computational study to investigate how AN, or p-AN groups compared to HCs use nested, local and global retrospective beliefs about HCT performance to update their explicit, prospective beliefs about their related abilities.

## Methods: Experiment 2

### Participants

This experiment had a non-overlapping sample to *Experiment 1* and the same eligibility (details on participant characteristics and recruitment sites in Supplementary Material). A total of N_AN_ = 40, N_p-AN_ = 40, and N_HC_ = 121 participants were screened. Following exclusions (see Supplementary Material), the final sample consisted of N_AN_ = 35, N_p-AN_ = 40, and N_HC_ = 117 participants (see Tables 1 and S8 for details on demographics and clinical characteristics).

### Belief Updating Task Design and Measures

**Figure 1:**
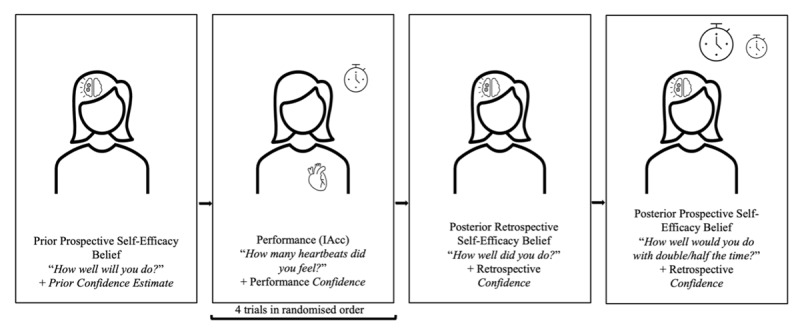
**Trial Timeline for the Heartbeat Counting Task.** The trial timeline shows the flow of the main Heartbeat Counting Task and the additional measures of *Experiment 2*, including our main experimental target: the updating of prospective self-efficacy (metacognitive) beliefs about heartbeat counting after trying out a heartbeat counting task and forming explicit retrospective beliefs about one’s performance. Specifically, we asked participants to tell us how well they think they would do if we gave them half the time, and then separately if we gave them double the time to complete the task. These questions allowed us to see how our participants used what they had observed and explicitly reported during the task (retrospective, metacognitive beliefs about their performance) to form self-efficacy beliefs that they could apply prospectively in a varied context.

Building upon the findings of *Experiment 1*, in *Experiment 2*, we examined how these self-efficacy beliefs are updated prospectively, when participants had to estimate their cardiac interoceptive abilities about a future HCT performance. Therefore, *Experiment 2* examined how AN and p-AN, compared to HC women, form and update Prospective Self-Efficacy Beliefs (see Figure 1 and below) about interoception before and after completing a modified version of the HCT (Schandry, 1981). We used the measure of Performance as in *Experiment 1*, and three additional self-efficacy measures, each with corresponding subjective confidence ratings. These measures included participants’ (1) Prior Prospective Self-Efficacy Beliefs (i.e., how well they think they will do on the HCT, as in *Experiment 1*), (2) Posterior Retrospective Self-Efficacy Beliefs (i.e., how well they think they performed on the task, as in *Experiment 1*), and (3) Posterior Prospective Self-Efficacy Beliefs (i.e., how well they think they would do in the future in the HCT; these self-efficacy beliefs about performance, explicitly sampled here for the first time, are identical to the sampled Prior Prospective Self-Efficacy Beliefs in that they require the participant to assess their future performance abilities) and hence allow us to examine how such prospective, global beliefs are updated after performance and local and global retrospective beliefs are generated; Figure 1). The Posterior Prospective Self-Efficacy Belief was the average of two scores: the first was a prospective rating of how well participants thought they would do if the four trials they had just completed were of half the duration in the future, while the second one asked them to rate how well they would do if the trials were of double the duration. In *Experiment 2* participants did not receive arbitrary false feedback (as they did in *Experiment 1*) and thus we did not obtain a measure of Post False-Feedback Retrospective Self-Efficacy Beliefs. We also introduced various control measures (see below).

### Primary Hypotheses, Measures and Analyses

#### 1. Behavioural

After *Experiment 1*, we examined why AN patients struggle to update their ‘pessimistic’ prospective self-efficacy beliefs despite a comparable performance to HCs on formal interoceptive tasks. Thus, the updating of these prospective, self-efficacy beliefs was the main measure of interest in *Experiment 2* and the primary focus of our computational modelling analyses (see below). As preregistered, we predicted that AN and p-AN participants would have lower Posterior Prospective Self-Efficacy Beliefs than HCs, despite predicting that we would find no evidence of group differences on Performance (as in *Experiment 1*).

To examine Group differences in Performance (calculated using the aforementioned Performance score transformation; see equation (1); Schandry, 1981), we ran a preregistered multilevel model analysis (MLM). As preregistered, we also controlled for knowledge about heartbeats, time estimation abilities, HRV and BMI, given criticisms of the effects of these aforementioned factors on Performance (Brener & Ring, 2016; Knapp-Kline & Kline, 2005; Richard et al., 2019; details in Supplementary Material).

In addition to a frequentist approach, we supplemented our analysis with a preregistered Bayesian analysis, which presents the ratio of the likelihood of the alternative hypothesis relative to the likelihood of the null hypothesis. A Bayes Factor (BF_10_) > 3 indicates evidence for the alternative hypothesis, whereas a BF_10_ < 3 indicates evidence for the null hypothesis. A BF_10_ between 0.3 and 3 indicates an inconclusive result which is not in favour of either hypothesis (Carey et al., 2021; Kass & Raftery, 1995).

Next, we tested between-group differences in self-efficacy beliefs after completing the HCT. To do this, and as preregistered, we assessed the effect of Group on Posterior Prospective Self-Efficacy Beliefs, using Age as a control variable, and Study Site as a random effect (given our multi-site testing, see Supplementary Material). In preregistered, exploratory analyses we tested whether differences in traits and behaviours often seen in the AN population and found in the present study (e.g., depression, and anxiety; Table 1), explained the group differences in self-efficacy beliefs (details in Supplementary Material, see Figures S1 and S2, and Table S11). The analyses were conducted following the Baron and Kenny (1986) mediation analysis steps, as outlined in detail in the Supplementary Material. In non-preregistered analyses we explored the role of set-shifting difficulties, as measured via the Wisconsin Card Sorting Test (WCST; Grant & Berg, 1948; results in Supplementary Material), on Posterior Prospective Self-Efficacy Beliefs. The WCST is used to assess cognitive flexibility and set-shifting: individuals need to categorise response cards based on different, shifting criteria, e.g., colour and shape (Kopp et al., 2021; Westwood et al., 2016).

**Table 1:** Participant Demographics and Characteristics for *Experiment 2*.


	AN	p-AN	HC	TEST STATISTIC [β, (*SE*)], *p*^1^	
			
	MEAN (*SD*)	MEAN (*SD*)	MEAN (*SD*)	AN vs HC	p-AN vs HC

N	35	40	117	–	–

Age	22(*6.19*)	26.51(*7.24*)	24.67(*5.76*)	–2.61(*1.19*), ***0.029***	1.84(*1.13*), *0.105*

BMI	16.13(*1.61*)	19.93(*1.82*)	20.48(*4.77*)	–4.35(*0.93*), ***<0.001***	–0.54(*1.05*), *0.604*

Illness Duration (years)	5.85(*6.39*)	7.29(*6.57*)	–	–	–

Heartbeats – Self*	65.94(*18.72*)	67.46(*18.28*)	68.94(*31.89*)	–3.00(*–1.48*), *0.684*	–1.48(*6.52*), *0.820*

Heartbeats at Rest*	69.29(*11.91*)	71.26(*9.44*)	73.03(*9.32*)	–3.74(*3.08*), *0.227*	–1.77(*2.34*), *0.450*

Heartbeats – Other*	60.11(*17.8*)	65.68(*16.76*)	68.72(*30.08*)	–8.60(*5.00*), *0.087*	–3.03(*4.85*), *0.533*

WCST – Percentage Correct	69.18(*14.66*)	74.45 (*14.85*)	72.76(*15.16*)	–3.58(*4.89*), *0.465*	1.69(*3.82*), *0.658*

WCST – Percentage Preservative	33.95(*8.84*)	30.76(*10.1*)	29.59(*10.69*)	4.35(*3.38*), *0.201*	1.16(*2.70*), *0.668*

EDE-Q Total	1.62(*1.42*)	1.44(*1.58*)	1.08(*1.07*)	0.55(*0.24*), ***0.026***	0.37(*0.23*), *0.116*

EDI-3 Interoceptive Deficits	14(*9.19*)	13.16(*10.15*)	9.5(*9.35*)	4.50(*1.85*), ***0.016***	3.66(*1.77*), ***0.040***

OCI-R Total	1.32(*0.79*)	1.13(*0.75*)	0.81(*0.62*)	0.051(*0.13*), ***0.001***	0.32(*0.13*), ***0.013***

IUS-12 Total	39.53(*12.23*)	34.82(*12.08*)	27.04(*9.36*)	12.49(*2.05*), ***<0.001***	7.77(*1.96*), ***0.001***

DASS-21 Depression	20.26(*12.92*)	14.00(*13.4*)	6.94(*7.01*)	13.32(*2.19*), ***<0.001***	7.06(*2.61*), ***0.008***

DASS-21 Anxiety	13.13(*9.96*)	11.87(*9.43*)	6.92(*6.55*)	6.21(*1.81*), ***0.001***	4.95(*2.15*), ***0.023***

DASS-21 Stress	22.87(*11.49*)	21.20(*10.92*)	11.13(*8.67*)	11.74(*2.25*), ***<0.001***	10.07(*2.68*), ***<0.001***

TAS-20 Total	56.03(*14.25*)	47.79(*13.87*)	42.24(*10.79*)	13.79(*2.36*), ***<0.001***	5.55(*2.26*), ***0.015***


N.B. Dashes indicate measure not taken. Heartbeats – Self*, and -Other* refer to participants’ estimation of their own resting heart rate, and their estimation of the average resting heart rate in the general population (beats per minute (bpm), respectively). Heartbeats at Rest refer to participants’ actual average bpm when at rest. Abbreviations: AN (Anorexia Nervosa); p-AN (post-acute Anorexia Nervosa); HC (Healthy Control); WCST (Wisconsin Card Sorting Task); EDE-Q (Eating Disorder Examination Questionnaire); EDI-3 (Eating Disorders Inventory 3); OCI-R (Obsessive Compulsive Inventory Revised); IUS (Intolerance of Uncertainty Scale); DASS-21 (21-Item Depression, Anxiety and Stress Scale); TAS-20 (20-Item Toronto Alexithymia Scale). ^1^Linear regressions were run to examine group differences, with HC as the intercept. As expected, we found between group differences on BMI and EDE-Q – such differences are axiomatic to our groups and consistent with our inclusion criteria. As we also observed expected group differences in psychometric traits, these were taken into account in our main analyses (see Results and Supplementary Material). We also found an unexpected group difference in age, and it was thus taken into account in our behavioural analyses on self-efficacy beliefs and Performance. Bolded values denote statistical significance (*p < 0.05*).

#### 2. Computational Modelling

The behavioural analyses were complemented with preregistered modelling analyses to account for the role of the nested nature of prior prospective and retrospective beliefs in the updating of such posterior prospective beliefs (see Introduction) and other parameters such as precision and learning rate. We first examined which model best predicted our key measure (Posterior Prospective Self-Efficacy Beliefs) by constructing and comparing between models that included the scores of different proxy-measures for evidence and for the precision of this evidence. We then compared how the winning model predicted our groups’ actual posterior prospective beliefs. Furthermore, we examined our clinical groups’ learning rate (using the winning model’s measures of ‘precision of prior beliefs’ and ‘precision of evidence’; see below), expecting it to be lower than the HCs’ when controlling for more general mental flexibility deficits. In a preregistered analysis, we then compared the learning rates expected by the equations of the winning model against the actual learning rates performed by the groups (i.e., absolute difference between the actual and precision-weighted learning rates) to assess if there are statistically significant group differences. Therefore, we were able to examine the Bayesian optimality of our groups’ learning rates based on the assumption – under a Bayesian belief updating mechanism – that an actual learning rate closer to the precision-weighted learning rate (where the latter describes the relative importance of evidence versus prior beliefs) is suggestive of a more Bayesian optimal learning (see below).

Specifically, we computed a posterior self-efficacy belief (*μ*_*θ*|_*_y_*) using a generic Bayesian equation for belief updating when receiving new information (or, evidence) under a Gaussian model with conjugate prior (Friston, 2017; Mathys et al., 2014; Kirsch et al., 2021)


2 \[
{\mu _{\theta |y}} = \,{\mu _\theta } + \frac{{{\pi _\varepsilon }}}{{{\pi _{\theta }}\, + {\pi _\varepsilon }}}\left({y - {\mu _\theta }} \right),
\]

where, *μ*_*θ*|_*_y_* was the posterior self-efficacy belief, *μ_θ_* was the prior prospective self-efficacy belief, *y* was the evidence (different measures per model; see below), *π_θ_* was the proxy for the precision of the prior prospective self-efficacy belief, and *π_ε_* was the proxy for the precision of the evidence (different measures per model; see below). Specifically, this equation allowed us to create a set of four target models to examine our hypotheses (see Supplementary Material Table S16 for full model description; and see Figures S7–S9 for model description and validation). For all four models we used participants’ Prior Prospective Self-Efficacy Beliefs as the prior (*μ_θ_*), and respective Prior Prospective Confidence estimates as a precision proxy of the prior (*π_θ_*). These models differed in the measures that were used as evidence (*y*; Performance versus Posterior Retrospective Self-Efficacy Beliefs) and as evidence precision proxies (*π_θ_*; Performance Confidence versus EDI-3-ID; Garner, 2004; the EDI-3-ID scores were rescaled as a success percentage rate to maintain consistency with the scoring of the other measures, e.g., Performance Confidence and Performance). For each model, we computed the Learning Rate (λ; also known as the Bayesian precision ratio) per participant:


3 \[
\lambda = \frac{{{\pi _\varepsilon }}}{{{\pi _{\theta }} + {\pi _\varepsilon }}}
\]

We also created two sets of baseline models (to validate our target models); the first two models assumed a perfect learning rate (λ =1, the participant uses the evidence as their posterior belief) and the third model assumed no learning (λ = 0, the participant uses the prior as their posterior belief). These baseline models were created as validation for the four main models, representing the boundary/extreme cases of learning rates being 1, or 0, instead of being modelled using the precision proxies for the prior and the evidence. They are presented in full in the Supplementary Material (Table S17).

For all the models we computed the Bayesian Information Criterion (BIC) and Mean Absolute Error (MAE) to measure model fit. As preregistered, we initially examined which variable was the best measure of evidence, in the HCs only, by comparing the fit of the model that used Performance Confidence as an evidence precision proxy and ‘Posterior Retrospective Self-Efficacy Beliefs’ as evidence with the alternative learning model that used the same precision proxy but Performance as evidence, predicting that the former model would show a better fit than the alternative learning model, particularly in the HCs (Prediction A). We repeated this across groups, and within each clinical group separately and in a non-preregistered analysis we explored the winning model’s validity (details in Supplementary Material and Table S18a).

For the measure of evidence which was associated with the best model fit from the previous analyses, we performed a preregistered further modelling step wherein we examined whether subjective confidence ratings (Performance Confidence) or trait measures of interoceptive sensibility (EDI-3-ID; Garner, 2004) when used as precision proxies, best captured our groups’ belief updating, expecting the clinical groups to be more influenced by the trait measures than HCs (Prediction B). We also complemented this precision-proxy comparison with two non-preregistered analyses to explore the winning model’s validity (details in Supplementary Material and Table S18b).

Next, we examined the between-group differences on precision-weighted Learning Rates. Assuming that the Bayesian Model of choice is a representation of actual learning, then a higher Learning Rate would suggest that participants consider the Prediction Error to a greater degree when updating their beliefs. We calculated one precision-weighted Learning Rate per precision proxy and looked at group differences in two separate analyses, expecting the clinical groups’ Learning Rates to be lower and less Bayesian optimal (i.e., greater absolute difference between participants’ actual and precision-weighted Learning Rates) than the HCs’ (preregistered Prediction C). Actual Learning Rates (*λ_Actual_*) were calculated using the following equation: *λ_Actual_* = (*μ_θ|y_*–*μ_θ_*)/(*y*–*μ_θ_*), (4) where *μ_θ|y_* represents participants’ Posterior Prospective Self-Efficacy Beliefs, *μ_θ_* represents participants’ Prior Prospective Self-Efficacy Beliefs, and *y* represents participants’ Posterior Retrospective Self-Efficacy Beliefs. Given the effect of Depression and Stress on self-efficacy beliefs (see behavioural results), in non-preregistered analyses we explored whether Depression and Stress scores mediated participants’ precision-weighted Learning Rates.

Finally, as preregistered, we examined group differences on actual Learning Rates, also expecting the Learning Rates of the clinical groups to be lower than those of the HCs (Prediction D; results from this analysis are presented in full in the Supplementary Material, Table S20).

### Experimental Materials and Procedure

Following baseline and demographic assessments (see below), participants were given an Empatica E4 watch (a medical-grade wearable device that records real-time physiological data; Empatica Srl, Italy; see https://www.empatica.com/research/e4/) to wear on their left wrist. The rest of the procedures were identical to those described in *Experiment 1* with the following exceptions. In addition to a 5-minute baseline recording of heart rate, we also obtained a recording of heart rate variability (HRV; used in *Experiment 2* as control measures; see Table 1, Supplementary Material and Table S3). The HCT instructions were the same, but here we also added one more counting phase of 35s, and after participants reported how many heartbeats they felt they also provided a confidence estimate (ranging from 0, *not at all confident* to 100, *extremely confident*) on the accuracy of each response (hereafter referred to as Performance Confidence). Participants also completed a time-estimation task before completing the heartbeat counting trials; they silently counted seconds until prompted to stop and then verbally reported how many seconds they counted (used in control analyses; see Supplementary Material). The duration of the time-estimation trials matched that of the heartbeat counting trials and they were also presented in a random order between participants.

At the end of the HCT participants reported how many heartbeats they think they typically have when at rest, and the general population average (per minute). The answers were used in control analyses (see Supplementary Material). Participants were not given any feedback on trial length or performance at any point. Finally, participants completed a series of psychometric questionnaires and cognitive flexibility task (see Supplementary Material) and were fully debriefed at the end.

## Results: Experiment 2

### Primary Hypotheses Results

#### HCT Performance did not differ significantly between our three groups

**Figure 2:**
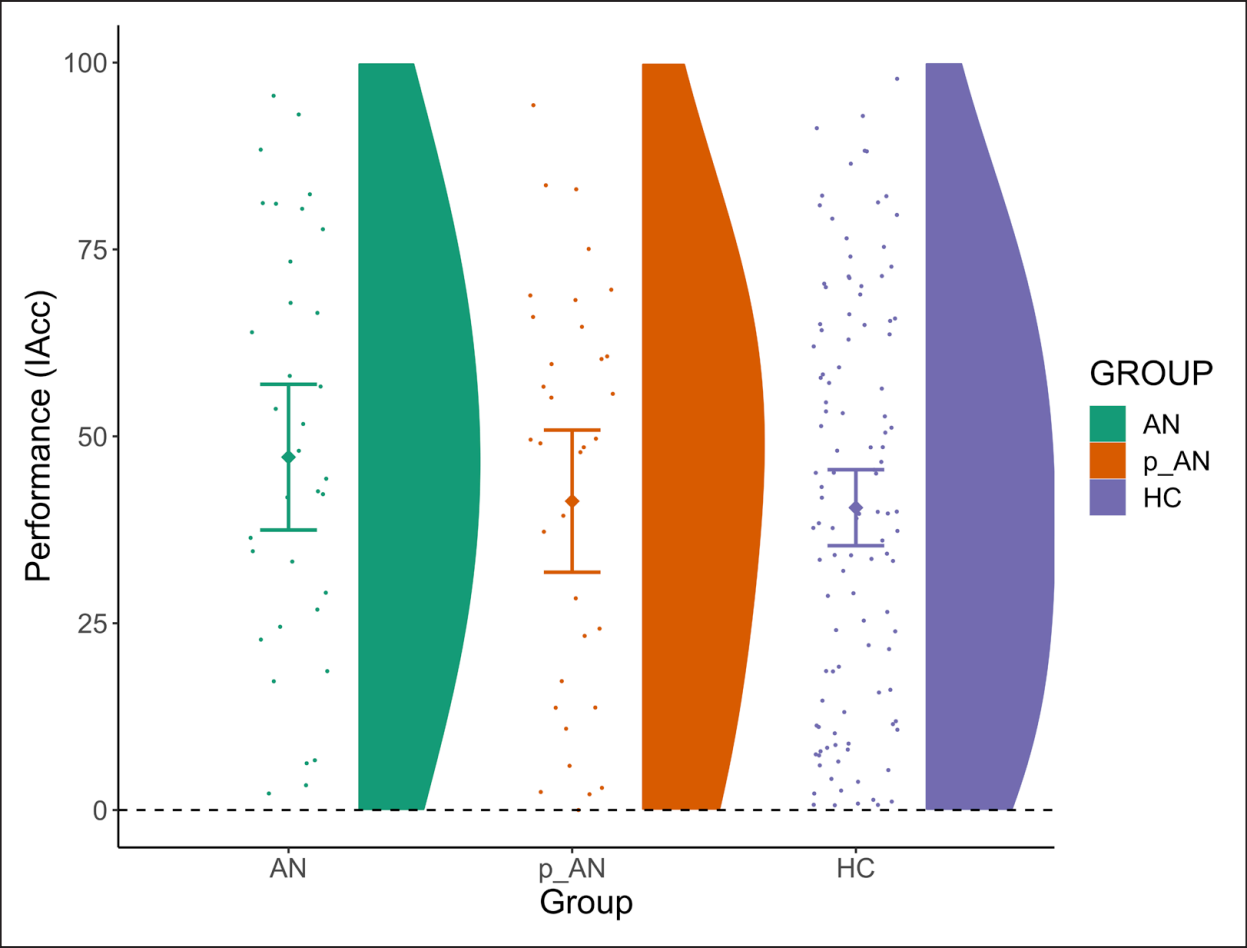
**Average Performance Scores at the heartbeat counting task (IAcc) per group.** N.B. We note the overall average Performance (IAcc) scores of our sample are lower than the average scores typically observed in other studies with both healthy and clinical populations (e.g., Desmedt et al., 2018; Kinnaird et al., 2020; Koreki et al., 2021), though it should be noted that they still fall within the spectrum of average IAcc scores observed when participants are encouraged to report only number of actually felt heartbeats and not estimates (e.g., Desmedt et al., 2018).

As predicted, the frequentist analysis did not yield a significant result (Figure 2; Table 2) on group differences in Performance. Moreover, the Bayes Factor analysis suggested that there is moderate evidence for equivalence regarding our groups’ Performance (BF_10_ = 0.28), indicating that as tested here, the AN and p-AN groups did not perform differently than HCs. We then ran preregistered control analyses to account for potential confounding variables and the pattern of results remained the same (see Supplementary Material and Table S10).

**Table 2:** Analysis for Group Differences on Performance, with Age as the Control Variable and Participant ID and Study Site as Random Effects.


*PREDICTORS*	β(*SE*)	*t*	95% CI	*p*

(Intercept)	50.05(*9.00*)	*5.56*	32.41 – 67.70	*<0.001*

AN	2.12(*5.72*)	*0.37*	–9.08 – 13.32	*0.711*

p-AN	0.36(*5.51*)	*0.07*	–10.43 – 11.14	*0.947*

AGE	–0.38(*0.33*)	*–1.16*	–1.04 – 0.27	*0.248*

**Random Effects**				

σ^2^	227.25			

τ_00 ID_	678.62			

τ_00 STUDY_SITE_	30.48			

ICC	0.76			

N_ID_	185			

N_STUDY_SITE_	4			

Observations	672			

Marginal R^2^/Conditional R^2^	0.008/0.759			


N.B. Bolded values denote statistical significance.

### Updating Prospective Beliefs: Behavioural and Computational Analysis

**Figure 3:**
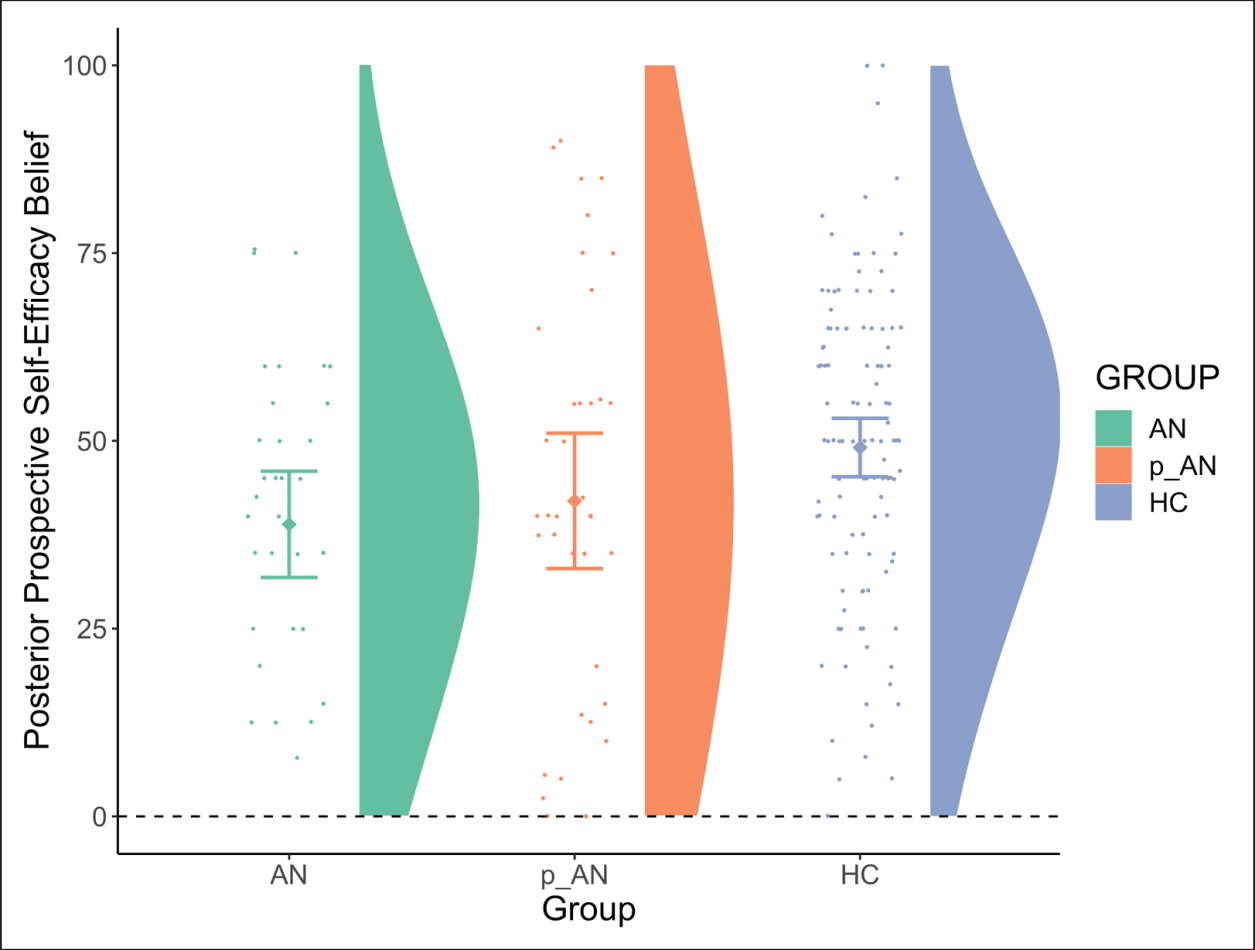
Average of Posterior Prospective Self-Efficacy Beliefs per group.

AN participants gave significantly lower Posterior Prospective Self-Efficacy Beliefs compared to HCs, and the same effect was present as a statistical trend in the p-AN group (Figure 3; Table 3). That is, both clinical groups expected, on average, to perform worse in a future HCT with half or double the available time, compared to HCs. To further examine what explained this observed ‘pessimism’ in our clinical groups, we examined the potentially mediating effect of comorbid traits and behaviours (using all the variables in which we found a significant group difference in Table 1). Only depression and stress, as measured via the Depression, Anxiety and Stress Scale (DASS-21; Lovibond & Lovibond, 1995) explained the clinical populations’ pessimistic Posterior Prospective Self-Efficacy Beliefs (see Supplementary Material and Table S11a for details and see follow-up, exploratory analyses below). We also explored whether a more general set-shifting difficulty could have explained the pessimistic beliefs of the clinical groups (in comparison to the HCs’) but found no significant effect of the WCST performance on Posterior Prospective Self-Efficacy Beliefs (Table S11b). We then examined, via computational modelling, the parameters which could affect the formation of these posterior prospective beliefs.

**Table 3:** Analysis for Group Differences on Posterior Prospective Self-Efficacy Beliefs, with Age as the Control Variable and Study Site as Random Effect.


*PREDICTORS*	β(*SE*)	*t*	95% CI	*p*

(Intercept)	50.25(*7.13*)	*7.05*	36.27 – 64.23	*<0.001*

AN	–10.31(*4.56*)	*–2.26*	–19.26 – –1.37	***0.024***

p-AN	–7.96(*4.34*)	*–1.83*	–16.47 – 0.55	*0.067*

AGE	–0.05(*0.27*)	*–0.20*	–0.59 – 0.48	*0.841*

**Random Effects**				

σ^2^	500.37			

τ_00 STUDY_SITE_	8.85			

ICC	0.02			

N_STUDY_SITE_	4			

Observations	189			

Marginal R^2^/Conditional R^2^	0.038/0.053			


N.B. Bolded values denote statistical significance.

### Computational Modelling

#### Identifying the best measure of evidence (Preregistered Prediction A)

**Figure 4:**
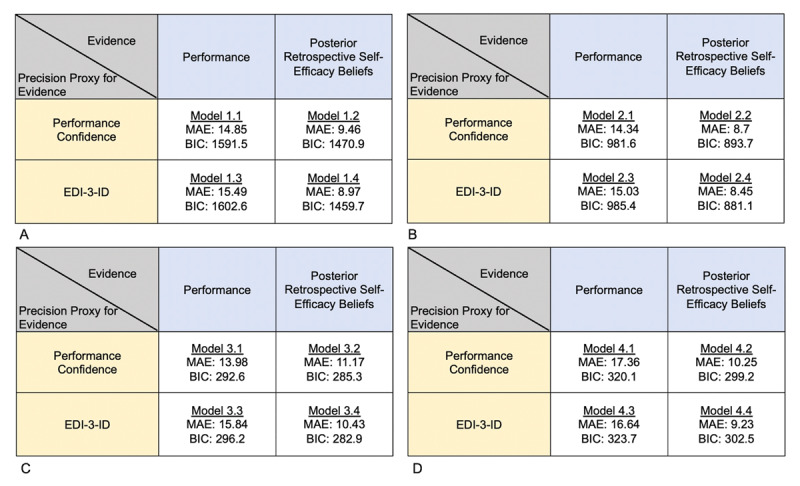
**Main model comparison.** MAE (Mean Absolute Error) and BIC (Bayesian Information Criterion) are two measures used to examine model fit, with smaller values suggesting better model fit. Panel A shows the model comparison across all participants (N = 183). Panel B shows the model comparison in the healthy control group only (N_HC_ = 114). Panel C shows the model comparison in the acute Anorexia Nervosa group only (N_AN_ = 34). Panel D shows the model comparison in the post-acute Anorexia Nervosa group only (N_p-AN_ = 35).

Firstly, we examined which model showed a better fit in HCs by comparing between two models that both used Performance Confidence as a precision proxy but differed in that Performance versus Posterior Retrospective Self-Efficacy Beliefs were used as alternative measures of evidence (Figure 4, Models 2.1 and 2.2). Model 2.2 was the winning model in HCs, but also across groups, and in each clinical group (Figure 4; Models 1.2, 3.2 and 4.2; see Supplementary Material and Table S18 for a non-preregistered parameter validation analysis).

#### Identifying the best measure for precision proxy of the evidence (Preregistered Prediction B)

In a further modelling step, we examined which precision proxies best captured our groups’ belief updating when Posterior Retrospective Self-Efficacy Beliefs were used as the evidence (winning model from above) and found that the model which used EDI-3-ID as a precision proxy (vs. our other competing precision proxy; Performance Confidence) was our winning model (both across groups and within each group; Figure 4, models 1.4, 2.4, 3.4, and 4.4), as predicted (see Supplementary Material and Table S18b for non-preregistered parameter validation analysis).

#### Group differences on precision-weighted Learning Rates and Bayesian Optimality with winning precision proxy of evidence (Preregistered Prediction C) and with the alternate precision proxy of evidence

Finally, for prediction C, we performed two further steps. Firstly, we looked at between-group differences in the precision-weighted Learning Rates (for both precision proxies of the evidence, given the inconclusive results of prediction B, but we Holm-corrected the *p* values due to the multiple comparisons). Secondly, we looked whether the Bayesian optimality of this rate, differed between groups, by assessing the absolute difference between our groups’ actual and precision-weighted Learning Rates (|*λ_Actual_*–*λ*|). Specifically, we ran two separate linear regressions to explore the Group effect on the precision-weighted Learning Rate. When using Performance Confidence as an evidence precision proxy to compute participants’ Learning Rates, we found that AN participants but not p-AN had a significantly lower Learning Rate than HCs (Table 4a; Prediction C). We found no significant between-group differences on Learning Rates when using EDI-3-ID as a precision proxy (Table 4a). We then ran two separate linear regressions to examine the optimality of the groups’ Learning Rates (calculated as the absolute difference between their actual and precision-weighted Learning Rates). However, when examining how the actual learning rates approximated the precision-weighted Learning Rate (with Performance Confidence as the precision proxy of the evidence), we did not find a significant difference (Table 4b). This result suggests that while we have significant group differences on precision-weighted Learning Rates, we cannot suggest that the learning rate mechanism of the AN patients is less Bayesian optimal than that of the HCs.

**Table 4:** Main output of Bayesian Belief Updating Analyses for *Experiment 2*.


4a: Precision-Weighted Learning Rate Comparisons				

**Learning Rate with Performance Confidence as a Precision Proxy**				

*Predictors*	β(*SE*)	*t*	95% CI	*p*

Intercept	0.47(*0.01*)	*31.53*	0.44 – 0.50	*<0.001*

AN	–0.09(*0.03*)	*–2.78*	–0.15 – –0.02	***0.012***

p-AN	–0.02(*0.03*)	*–0.76*	–0.08 – –0.04	*0.446*

**Learning Rate with EDI-3-ID as a Precision Proxy**				

Intercept	0.52(*0.02*)	*33.53*	0.49 – 0.55	*<0.001*

AN	–0.03(*0.03*)	*–0.93*	–0.09 – 0.03	*0.704*

p-AN	–0.01(*0.03*)	*–0.41*	–0.07 – 0.05	*0.704*

4b: Comparisons to Examine the Bayesian Optimality of the Precision-Weighted Learning rates				

Absolute Difference Between Actual Learning Rate and Learning Rate 1*				

(Intercept)	0.59(*0.05*)	*11.07*	0.49 – 0.70	*<0.001*

AN	0.16(*0.12*)	*1.42*	–0.06 – 0.39	*0.159*

p-AN	–0.01(*0.11*)	*–0.07*	–0.22 – 0.21	*0.946*

Absolute Difference Between Actual Learning Rate and Learning Rate 2*				

(Intercept)	0.57(*0.05*)	*10.80*	0.47 – 0.68	*<0.001*

AN	0.12(*0.12*)	*1.03*	–0.11 – 0.35	*0.306*

p-AN	–0.03(*0.11*)	*–0.23*	–0.24 – 0.19	*0.817*

4c. Mediation Analyses to Explore the Role of Depression and Stress on Learning Rates**				

**Analysis on (precision-weighted) Learning Rates using Depression and Stress as the Predictors (Mediation Analysis Step 2)**				

(Intercept)	0.45(*0.01*)	*33.32*	0.42 – 0.47	*<0.001*

Depression	–0.03(*0.01*)	*–2.62*	–0.06 – –0.01	***0.010***

Stress	–0.05(*0.01*)	*–3.85*	–0.08 – –0.03	***<0.001***

**Analysis on (precision-weighted) Learning Rates using Group, Depression and Stress as the Predictors (Mediation Analysis Step 3)**				

(Intercept)	0.46(*0.02*)	*27.28*	0.43 – 0.50	*<0.001*

AN	–0.07(*0.04*)	*–1.88*	–0.14 – 0.00	*0.063*

p-AN	–0.05(*0.04*)	*1.15*	–0.13 – 0.04	*0.252*

Depression	0.03(*0.01*)	*–1.81*	–0.05 – 0.00	*0.073*

Stress	–0.04(*0.01*)	*–3.08*	–0.07 – –0.02	***0.003***

**Holm-corrected analysis on (precision-weighted) Learning Rates of the AN group using Depression and Stress as the Predictors**				

(Intercept)	0.38(*0.05*)	*7.02*	0.27 – 0.50	*<0.001*

Depression	–0.00(*0.04*)	*–0.09*	–0.08 – 0.08	*0.933*

Stress	–0.06(*0.04*)	*–1.25*	–0.15 – 0.04	*0.675*

**Holm-corrected analysis on (precision-weighted) Learning Rates of the p-AN group using Depression and Stress as the Predictors**				

(Intercept)	0.38(*0.03*)	*11.04*	0.30 – 0.45	*<0.001*

Depression	–0.09(*0.03*)	*–3.32*	–0.14 – –0.03	***0.024***

Stress	0.04(*0.04*)	*0.95*	–0.05 – 0.12	*0.362*

**Analysis on Performance Confidence using Depression and Stress as the Predictors (Mediation Analysis Step 2)**				

(Intercept)	53.43(*2.12*)	*25.21*	49.23 – 57.63	<0.001

Depression	–5.98(*2.08*)	*–2.08*	–10.10 – –1.87	**0.005**

Stress	–6.78(*2.13*)	*2.13*	–10.99 – –2.56	**0.002**

**Analysis on Performance Confidence using Group, Depression and Stress as the Predictors (Mediation Analysis Step 3)**				

(Intercept)	56.45(*2.60*)	*21.68*	51.30 – 61.51	*<0.001*

AN	–8.72(*5.94*)	*–1.47*	–20.49 – 3.05	*0.145*

p-AN	–11.39(*6.72*)	*–1.69*	–24.70 – 1.93	*0.093*

Depression	–4.81(*2.20*)	*–2.19*	–9.16 – –0.45	***0.031***

Stress	–5.35(*2.23*)	*–2.40*	–9.76 – –0.94	***0.018***

**Analysis on actual Learning Rates using Depression and Stress as the Predictors (Mediation Analysis Step 2)**				

(Intercept)	0.71(*0.07*)	*10.76*	0.58 – 0.84	*<0.001*

Depression	–0.12(*0.06*)	*–1.83*	–0.25 – 0.01	*0.070*

Stress	0.06(*0.07*)	*0.88*	–0.08 – 0.20	*0.379*

**Analysis on the absolute difference between actual and precision-weighted Learning Rates using Depression and Stress as Predictors (Mediation Analysis Step 2)**				

(Intercept)	0.60(*0.05*)	*11.05*	0.49 – 0.71	*<0.001*

Depression	0.06(*0.05*)	*1.19*	–0.04 – 0.17	*0.238*

Stress	–0.05(*0.06*)	*–0.95*	–0.17 – 0.06	*0.347*


N.B. Bolded values denote statistical significance (*p* < *0.05*). * Learning Rate 1 refers to the precision-weighted learning rate with Performance Confidence as a proxy for precision of the evidence; Learning Rate 2 refers to the precision-weighted learning rate with EDI-3-ID as a proxy for precision of the evidence. **Here we only examined the role of Depression and Stress on the precision-weighted Learning Rates which used Performance Confidence as a precision proxy of evidence, given that we only found significant group differences on these learning rates and not the ones computed using EDI-3-ID as a precision proxy of evidence.

#### Exploring the Role of Depression and Stress

Given the effects of Depression and Stress on the clinical groups’ Posterior Prospective Self-Efficacy Beliefs (see Supplementary Material and Table S11) as well as the group differences on these psychometric trait measures (step 1 of the mediation analysis; see results in Table 1 and mediation analysis steps in the Supplementary Material), in non-preregistered analyses we explored whether Depression and Stress also explained the difference between the AN and HC groups’ precision-weighted Learning Rates (when using Performance Confidence as an evidence precision proxy). First, we ran a linear regression using Depression and Stress as predictor variables (step 2 of the mediation analysis; see Supplementary Material): Learning Rate lowered as Depression and Stress scores increased (Table 4c). We then explored whether Depression and Stress explained the group effect (step 3 of the mediation analysis; see Supplementary Material): we found a non-significant trend of Depression and a significant effect of Stress on Learning Rates, which would explain the difference between the AN and HC groups’ Learning Rates. We complemented with Holm-corrected linear regressions on the AN and p-AN groups separately, using Depression and Stress scores as predictors to examine trait and state effects. Higher Depression scores significantly influenced the p-AN group’s Learning Rates. Although we found a non-significant effect on the AN group, we suggest this is due to the already high Depression scores within the entire sample (same as in the self-efficacy beliefs) which we discuss in detail in the Discussion. Next, given that the precision-weighted Learning Rates were computed using Performance Confidence as a precision proxy of evidence and that the AN group had a lower Performance Confidence than HCs (Table S15b), we also examined whether Depression and Stress mediated the group differences on Performance Confidence. Higher Depression and Stress scores explained lower Performance Confidence Estimates. This suggests that as the uncertainty on the evidence increases, there is less evidence- and more prior-weighting, explaining the observed lower Learning Rate of the AN group in comparison to that of HCs. For control purposes we examined whether these findings also applied when looking at the effects of Depression and Stress on the actual Learning Rates and not only on the modelled, precision-weighted Learning Rates, but we found a non-significant trend of Depression scores on actual Learning Rates, and no significant effect of Stress. Although Depression and Stress influenced the precision-weighted Learning Rates, they did not significantly influence the Learning Rates’ Bayesian optimality (Table 4c).

## General Discussion

We examined how patients in the acute and post-acute anorexia nervosa phase (AN and p-AN, respectively), compared to HCs update their self-efficacy beliefs about their heartbeat counting abilities. In *Experiment 1*, AN patients showed lower self-efficacy beliefs than HCs before (prospectively) and after the task (retrospectively), despite performing comparably to HCs. Our preregistered *Experiment 2* aimed to examine specifically how such pessimistic prospective beliefs are formed and maintained. As predicted, although AN patients performed comparably to HCs, they were more pessimistic than HCs when asked how they would do in a similar task in the future. Furthermore, computational analyses, revealed that AN patients seem to rely more on their pessimistic retrospective beliefs about interoceptive performance rather than their actual performance when updating their beliefs prospectively. AN patients also show low confidence in the accuracy of their performance, which when accounted for reveals a smaller learning rate in AN patients than controls, indicating that they make less use of prediction errors. Interestingly, the critical parameters revealed by our computational analyses were associated more with mood than with cognitive traits.

As expected, our groups did not perform differently to each other in the actual HCT (also supported by Bayesian equivalence testing) in either experiment (and in line with most studies of similar populations using the HCT; e.g., Kinnaird et al., 2020; Lutz et al., 2019; Fischer et al., 2016; although contrary findings also exist, e.g., Pollatos et al., 2008, 2016). Study discrepancies may stem from the HCT’s noted low validity and reliability and its confounds (Brener & Ring, 2016; Desmedt et al., 2018, 2020, 2022; Legrand et al., 2022). For example, performance (interoceptive accuracy) may differ depending on task demands, time estimation abilities and heartbeat knowledge. Here, control analyses on this confounding variables (see Supplementary Material) suggest these factors are unlikely to have influenced Performance in our sample (in line with Ferentzi et al., 2022). Sampling differences (e.g., comorbidities, treatment type, illness stage; Fischer et al., 2016; Pollatos et al., 2008; Richard et al., 2019) could also account for the between-study differences on interoceptive accuracy. For example, here we only included individuals who met restrictive AN criteria and had no other ED diagnosis (American Psychiatric Association, 2013), unlike others. Notwithstanding heterogeneity in treatment stage and type, BMI and clinical profiles did not affect our findings (see Supplementary Material), suggesting that these factors were unlikely to have influenced our sample’s Performance. Despite controlling for some of the possible task confounds, future studies on interoceptive metacognition in AN could use the more recently developed tasks to capture interoceptive accuracy (see Desmedt et al., 2023; Garfinkel et al., 2022; Harrison et al., 2021; Legrand et al., 2022; Palmer et al., 2019; Plans et al., 2021), or use pharmacological (Khalsa et al., 2009, 2015) or behavioural (Fitz-Clarke, 2007; Smith, Feinstein et al., 2021) heart rate manipulations to increase the signal strength. We note however, that as our emphasis here was on testing explicit belief updating and not interoceptive accuracy *per se*, the HCT has the advantage of good patient acceptability and was easily understood as a brief task that one can have meaningful self-efficacy beliefs about. These self-efficacy beliefs were focused on participants’ interoceptive abilities (i.e., ‘*How well will you feel your heartbeat*’), rather than self-efficacy beliefs about everyday scenarios as typically assessed via questionnaires (e.g., “*If I am in trouble I can usually think of a solution*”; General Self-Efficacy Scale; Schwarzer & Jerusalem, 1995).

A key finding here is that AN patients (and p-AN patients at trend levels) do not seem to place sufficient emphasis on their performance when forming prospective self-efficacy beliefs. Hitherto, questionnaire studies consistently reveal that AN patients are characterised by higher levels of worry, rumination, and other maladaptive, metacognitive beliefs (e.g., Berman, 2006; Davenport et al., 2015; Palmieri et al., 2021), but these studies have not examined beliefs about interoceptive accuracy, or the various interwoven parameters that may underlie these beliefs, as done in our computational modelling. Moreover, we demonstrated that our experimental measure of ‘Posterior Prospective Self-Efficacy Beliefs’ was associated with beliefs about everyday life, such as fear of gaining weight, and less hope for symptom improvement. These findings suggest that our task had good ecological validity and examining computationally the various parameters that influence such beliefs in our task could provide insights regarding patients’ everyday negative metacognitive beliefs about interoception.

Specifically, our study leads to three main insights regarding such beliefs. First, AN patients’ updating of explicit, prospective beliefs was influenced more by “pessimistic” (i.e., lower) global retrospective beliefs about such performance (e.g., how accurate one thought they were after the task ends) rather than the perceptual performance itself (e.g., how accurate one was), as detailed below. Specifically, model comparisons revealed that retrospective beliefs were better at predicting posterior prospective beliefs than performance, with the winning model (the one using retrospective beliefs as evidence) being better at predicting the posterior prospective beliefs of the HCs than those of our clinical populations. This observation was also supported by poorer retrospective, interoceptive awareness (as indexed by the Interoceptive Trait Prediction Error (ITPE) z-score analyses; see Supplementary Material) in the clinical groups, compared to HCs who evaluated their performance as better than it was. Moreover, the AN had lower Performance Confidence than HCs, and combined with lower, explicit global retrospective beliefs about performance, this suggests that AN patients struggle more than HCs in incorporating new evidence (from the HCT) to update beliefs retrospectively. This pattern of findings suggests that irrespective of any individual differences in interoceptive accuracy *per se*, AN patients may face difficulties in drawing metacognitive conclusions about their cardiac interoceptive performance retrospectively.

Moreover, these retrospective metacognition difficulties appear to extend to prospective metacognition. Specifically, when participants needed to then use such retrospective beliefs to update their self-efficacy prospectively, AN patients showed lower precision-weighted Learning Rates (with Performance Confidence as a precision proxy of evidence) than HCs, suggesting that patients consider the ‘evidence’ (here, Posterior Retrospective Self-Efficacy Beliefs) less than HCs when updating such beliefs. Consequently, it is plausible that AN patients rely more on prior prospective beliefs regarding their cardiac interoceptive abilities, and this ultimately influences their explicit, global (posterior) prospective beliefs. Therefore, neither local evidence from performance, nor retrospective beliefs following that performance (that themselves were poorly updated by prediction errors, and hence may be hard to target therapeutically) seem to be sufficient to counter and update pessimistic, self-efficacy beliefs in AN. Instead, it may be the ‘precision-based’, mechanism of belief updating itself that requires therapeutic targeting. Indeed, it has been previously hypothesised in two, transdiagnostic studies (Lavalley et al., 2023; Smith, Kirlic, et al., 2021; see Introduction) that an inappropriate weighting of prior beliefs versus new evidence, especially regarding interoception, may lead to the manifestation of several psychiatric symptoms (Barca & Pezzulo, 2020; Barrett & Simmons, 2015; Owens et al., 2018; Paulus et al., 2019; Petzschner et al., 2017). Indeed, while pessimistic estimates about interoceptive abilities have been documented by existing studies on an anticipatory and perceptual level (e.g., Crucianelli et al., 2016, 2021; Khalsa et al., 2015; Lutz et al., 2019), few have envisioned faulty updating at metacognitive, and prospective cognition levels (e.g., Stephan et al., 2016).

Second, we explored the potential role of other higher-order cognitive difficulties previously noted in AN, such as disruptions in mental flexibility, abstraction or set-shifting (Lang et al., 2014; Miles et al., 2020, 2022; Tchanturia et al., 2012), which could have explained the reduced belief updating in AN. However, we found that cognitive, set-shifting abilities as tested here by the Wisconsin Card Sorting Task (WCST) in a sample subset did not explain group differences in posterior beliefs or learning rates. Indeed, prior studies have shown that such domain general, ‘frontal’ functions cannot explain some of the delusionality in AN (see Konstantakopoulos et al., 2011; 2012 for delusional body image beliefs and insight correlates in AN; see also Introduction), and *Experiment 1* showed that AN patients can update their beliefs based on external (albeit random) feedback (see also Kube et al., 2022). Notwithstanding, our study did not systematically test belief updating across different modalities and thus no conclusions about the interoceptive or more general domain-specificity of our findings are warranted.

Third, depression and stress scores predicted pessimism in self-efficacy beliefs, explaining why the clinical groups who notably had greater depression and stress levels also had more pessimistic Posterior Prospective Self-Efficacy Beliefs compared to HCs. Crucially, depression (but not stress) significantly influenced the p-AN group’s (but not the AN’s) posterior self-efficacy beliefs (i.e., the more severe the depression, the more pessimistic the beliefs). The AN group was overall more pessimistic and had more severe depression and stress than the HCs, making it difficult to disentangle which of these factors is more predictive of their lower self-efficacy beliefs, but our results showed the overall effect of depression and stress across all groups. Depression, anxiety and stress are key comorbidities with EDs, including AN (Andrés-Pepiñá et al., 2020; Eskild-Jensen et al., 2020; Himmerich et al., 2019); our findings also support previously noted associations between depression, negative metacognitive thoughts, and ED symptoms (e.g., Cooper et al., 2007; Palomba et al., 2017; see Introduction). Notably, some research traditions could interpret these findings as though negative mood could ‘explain away’ the pessimistic beliefs about interoception, from a more transdiagnostic, computational psychiatry perspective, our study can elucidate the mechanisms by which depression and stress symptoms can influence belief updating (e.g., Aylward et al., 2020; Katyal et al., 2023; Kim et al., 2020; Rupprechter et al., 2018; Stankevicius et al., 2014). Specifically, we explored if and how these two traits explained the observed group effect on the precision-weighted learning rates (when using Performance Confidence as a precision proxy of evidence). Interestingly, higher uncertainty on the precision of the evidence (as indexed by lower Performance Confidence estimates) was influenced by higher depression and stress scores, which in turn also explained the lower precision-weighted learning rates of the AN group compared to HCs, adding to previously found associations between local confidence and mood disorder symptom severity (Benwell et al., 2022; Rouault, Seow et al., 2018; Seow et al., 2021). Our findings, however, suggest that mood may relate to a dysfunctional precision-weighting mechanism extending beyond local metacognition to global, prospective beliefs about one’s performance. Moreover, interestingly, a recent theoretical framework has posited depression as a consequence of chronic dysregulation in interoception, suggesting a chronic low self-efficacy regarding homeostatic/allostatic control (Stephan et al., 2016). Although our study was cross-sectional and did not target interoceptive control specifically (i.e., we cannot characterise the hypothesised chronic mechanisms here), the association between depressive symptoms and pessimistic posterior beliefs of the p-AN group compared to controls (at trend-level), suggests that this dimension of illness may be an enduring AN trait, beyond the acute state.

In addition to some of the aforementioned experimental methodology limitations, there were also clinical methodology limitations. Firstly, multi-site research is subject to between-site variability relating to different assessor, clinical, cultural, and practical restrictions. To address this, meticulous protocol translations and experimenter training were ensured, and study site as a random effect did not add significant variance or affect the main results. Moreover, the multi-site approach could be regarded as a strength of our study, in line with recent efforts for more representative and diverse samples (Naddaf, 2023). Moreover, patient populations also had varying pharmacotherapy based on national guidelines, making standardised control for its effects beyond assessment via self-report questionnaires and medical records impossible. However, future studies should consider controlling for the role of pharmacotherapy in belief updating, in line with evidence on the effects of neuromodulators (e.g., dopamine and serotonin) in prediction errors, active inference, and precision-weighting mechanisms (Auksztulewicz & Friston, 2016; Daw & Doya, 2006; Haarsma et al., 2021; Iglesias et al., 2013; Schultz et al., 1997). Finally, the study focussed mostly on white women with AN due to the availability of these patients in the collaborating clinics. We suggest that future research should consider more diverse populations when studying interoception in AN, while accounting for the noted sex differences in AN diagnostic criteria, hormones and subsequent effect on interoception (e.g., Culbert et al., 2021; Gorrell & Murray, 2019; Grabauskaitė et al., 2017; Murphy et al., 2019; Suschinsky & Lalumière, 2012).

## Conclusion

To conclude, we investigated explicit, interoceptive belief updating in AN, focussing on cardiac awareness and related, prospective self-efficacy beliefs using a computational Bayesian Learning Framework. Despite comparable HCT performance, AN participants failed to update their interoceptive, metacognitive self-efficacy beliefs after performance. Computational modelling showed that neither local evidence from performance, nor retrospective beliefs following that performance (that themselves were suboptimally updated) seemed to be sufficient to counter and update pessimistic, self-efficacy beliefs in AN. AN patients showed lower learning rates than controls, revealing a tendency to base their posterior beliefs more on prior beliefs rather than prediction errors in both retrospective and prospective belief updating. These differences in both explicit beliefs, and the latent mechanisms of belief updating, were not explained by cognitive inflexibility, but by depression and stress measures, even after the acute AN phase. Our findings offer novel insights regarding clinically relevant, metacognitive belief updating difficulties about interoception in acute AN and their relation to depression and stress. However, future studies should aim to further explore belief updating across different modalities and domains, and in longitudinal designs, to draw conclusions regarding the specificity of these belief update difficulties.

## Additional File

The additional file for this article can be found as follows:

10.5334/cpsy.109.s1 Supplementary Material. Supplemental Text, Supplemental Methods, Supplemental Results, Supplemental Figures and Tables.
