# Supplementary material for: Updating Prospective Self-Efficacy Beliefs About Cardiac Interoception in Anorexia Nervosa: An Experimental and Computational Study
Source: Comput Psychiatr. 2024 Jun 26;8(1):92–118. doi: 10.5334/cpsy.109 (PMC11212784; doi:10.5334/cpsy.109)
Supplement: Supplementary Material. — Supplemental Text, Supplemental Methods, Supplemental Results, Supplemental Figures and Tables. [file cpsy-8-1-109-s1.pdf]

Updating Prospective Self-Efficacy Beliefs About Cardiac Interoception in Anorexia Nervosa: An Experimental and Computational Study

Alkistis Saramandi[1*], Laura Crucianelli[1,2*], Athanasios Koukoutsakis[1], Veronica Nisticò[1,3,4,5], Liza Mavromara[1,6], Diana Goeta[7], Giovanni Boido[3], Fragiskos Gonidakis[6], Benedetta Demartini[1,3,4,8], Sara Bertelli[8], Orsola Gambini[3,4,8], Paul M Jenkinson[1], Aikaterini Fotopoulou[1]

[1]Department of Clinical, Educational and Health Psychology, University College London, UK

[2]Department of Biological and Experimental Psychology, Queen Mary University of London, London, UK

[3]Department of Health Sciences, University of Milan, Milan, Italy

[4] Aldo Ravelli Research Centre for Neurotechnology and Experimental Brain Therapeutics, University of Milan, Italy

[5] Department of Psychology, University of Milan-Bicocca, Milan, Italy

[6] Eating Disorders' Unit, 1st Department of Psychiatry, National and Kapodistrian University of Athens, Greece

[7] Psychiatry Unit, ASST Santi Paolo e Carlo, S. Carlo General Hospital, Milan, Italy

[8] Psychiatry Unit, ASST Santi Paolo e Carlo, S. Paolo General Hospital, Milan, Italy

Corresponding Author:
Alkistis Saramandi, Miss
Department of Clinical, Educational, and Health Psychology
University College London (UCL)
Gower Street, WC1E 6BT
London, United Kingdom

Email: zcjtasa@ucl.ac.uk

*Joint first authorship

Supplementary Material

Table of Contents

Supplementary Material 2
Experiment 1: Supplementary Methods 4
Participants 4
Psychometric Measures 7
Data Analysis 9
Experiment 1: Supplementary Results 10
Control analyses for Prior Prospective Self-Efficacy Beliefs and Performance 10
Control analysis for Post False-Feedback Retrospective Self-Efficacy Belief 11
Analyses with Psychometric Traits and Items from the Insight into Illness Questionnaire 12
Supplementary Methods: Experiment 2 17
Participants 17
Psychometric Measures 18
Secondary Hypotheses, Measures and Analyses 21
Supplementary Results: Experiment 2 25
1.1 Main Measures (Performance and Posterior Prospective Self-Efficacy Beliefs) – Control and Exploratory Analyses 25
1.2 Secondary Measures Analysis Results 31
1.3 Computational Modelling: Model Presentation and Comparisons, Control and Exploratory Analyses 37
1.4 Exploratory Analyses in the acute and post-acute AN groups 45

Outline

In this Supplementary Material we provide further information about *Experiments 1* and *2*. The first section presents additional information for the Methods and Results of *Experiment 1*. Specifically, we present additional information about recruitment sites, participants' characteristics and clinical profiles, including comorbidities and pharmacological treatment (Supplemental Methods: *Experiment 1*, *Participants* section and Table S1), as well as details on questionnaires we used to assess psychological traits often observed in patients with Anorexia Nervosa (AN; *Methods, Psychometric Measures* and Table S2). We also offer more details on our data analysis plan and present the results from the control and secondary, exploratory analyses (Supplemental Results: *Experiment 1*, Tables S3 – S7). Next, the second main section offers supplemental information for the Methods and Results of *Experiment 2*. In particular, we present additional information about our sample (as in *Experiment 1*; see Supplemental Methods; *Experiment 2*, and Table S8), details on our secondary aims and hypotheses and respective data analyses plan, and a list of the questionnaires that were administered to assess psychological traits often observed in patients with AN (*Psychometric Measures* section, and Table S9). The Supplementary Results section for *Experiment 2* presents the results from our additional analyses on our main measures, the results from our analyses on our secondary hypotheses and measures, and the results from our exploratory analysis. Specifically, in the first part, the results from the additional, preregistered analyses for Performance (Table S3) and Posterior Prospective Self-Efficacy Beliefs (Table S4) are presented. In the second part, we present the results from the preregistered analyses on our secondary measures. First, we present the results from the analyses on Prior Prospective Self-Efficacy Belief group differences (Table S10). Then, we present the histograms for the Performance Error Percentage scores (Figures S4-S6) to demonstrate why we ran a further non-preregistered analysis between participants' Posterior Retrospective Self-Efficacy Beliefs and Performance Scores, as well as the results of the respective statistical analyses (Table S13). We then present the results from our preregistered analyses on Interoceptive Awareness and participants' Interoceptive Trait Prediction Error (ITPE) scores (Table S14), followed by group differences on Confidence Estimates (Table S15). In the third part of the Supplementary Results section for *Experiment 2*, we present the preregistered target models we created in detail (Table S16), the comparisons between the baseline models we created for our analyses under the Bayesian Learning Framework (Table S17) and the non-preregistered analyses we ran to test whether our winning model was indeed significantly better than the other competing models (Table S18 to complement the results presented in Figure 4), as well

as the winning model description and validation (Figures S7 – S9). We also present the results from our non-preregistered analyses using the percentage of correct WCST answers, and the percentage of perseverance on the WCST as a random effects to examine whether set-shifting abilities influenced participants' Learning Rate (Table S19). Finally, we ran preregistered analyses on participants' actual Learning Rates to examine potential between-group differences (Table S20). In the fourth and final part of the Supplementary Results section, we present the results from our preregistered exploratory analyses on self-efficacy beliefs and clinical symptoms in the clinical populations (Tables S21 – S23).

Experiment 1: Supplementary Methods

Participants

AN patients met the restrictive subtype AN DSM-5 criteria (American Psychiatric Association, 2013) as diagnosed by an experienced clinician using standard clinical measures (patient history, medical files, psychometric questionnaires - these include the Hamilton Anxiety and Depression Rating Scales (Hamilton, 1959, 1960), the Beck Anxiety Inventory (Beck & Steer, 1990) to assess further comorbidities and behaviours - and a physical assessment). There were no menstrual cycle criteria, consistent with the DSM-5 (American Psychiatric Association, 2013). Participants were eligible if they had no history of brain damage or disease, drug dependence and/or severe psychiatric disease and neurodevelopmental disorders (e.g., schizophrenia and symptoms of psychosis, hallucinations, and/or delusions, bipolar disorder, autism spectrum disorder). Other exclusion criteria included pregnancy, being male, and any report or indication of current substance and/or alcohol abuse.

The recruitment and testing for Experiment 1 took place in Italy and the UK. Participants from the AN and p-AN groups were recruited from the Psychiatric Daycare Unit of San Paolo General Hospital (ASST Santi Paolo e Carlo) in Milan, from Comunità Villa Miralago in Cuasso al Monte (VI) and from Casa di Cura Villa Margherita in Arcugnano, Italy. The HC participants were Italian volunteers recruited from the University of Milan (Italy) and University College London (UK). Local institutional ethics approval was obtained, and participants received either monetary compensation at a fixed rate of £7.5/hour, or university course credits (if in the p-AN or HC group), or £10 Amazon vouchers (if in the AN group).

Table S1. *Experiment 1 Participant Profiles and Clinical Characteristics*

	AN	p-AN	HC	Test Statistic [β, (SE)], *p*[1]	
	Mean (*SD*)	Mean (*SD*)	Mean (*SD*)	AN vs HC	p-AN vs HC
N	51	47	63	-	-
Age	26.51 (*9.48*)	25.38 (*6.23*)	25.14 (*4.77*)	1.37(1.31), *0.299*	0.24(1.34), *0.858*
BMI	15.80 (*1.53*)	19.89 (*2.46*)	21.17 (*2.98*)	-5.37(0.47, <***0.001***	-1.29(0.47), ***0.008***
Average heartbeat *	45.80(*8.60*)	47.59(*11.45*)	66.40(*18.24*)	-20.60(*3.10*), ***<0.001***	-18.81(*3.18*), ***<0.001***
Illness Duration (years)	8.21 (*8.45*)	4.36 (*3.85*)	-	-	-
Age of Onset	16.65 (*3.54*)	17.59 (*4.24*)	-	-	-
Years from Weight Restoration	-	3.47 (*5.40*)	-	-	-
DASS-21 Depression	12.85(*5.47*)	7.65(*6.00*)	3.31(*3.69*)	9.54(1.28), <***0.001***	4.34(1.34), ***0.002***
DASS-21 Anxiety	7.33(*4.52*)	6.04(*4.99*)	2.29(*2.35*)	5.05(1.01), <***0.001***	3.76(1.05), ***0.001***
DASS-21 Stress	13.04(*4.59*)	8.61(*5.29*)	6.34(*4.27*)	6.69(1.19), <***0.001***	2.27(1.25), *0.074*
BAQ Total	72.08(*18.00*)	74.21(*17.86*)	81.50(*14.82*)	-9.42(4.77), ***0.053***	-7.29(4.88), *0.140*
TAS-20 Total	61.53(*16.17*)	48.24(*16.08*)	39.66(*9.88*)	21.87(2.66), <***0.001***	8.58(2.74), ***0.002***

Psychiatric Comorbidities	5 OCD<br>7 Mood Disorder<br>3 Panic/Anxiety Disorders<br>4 Personality Disorder<br>15 Multiple Comorbidities	2 OCD<br>8 Mood Disorder<br>10 Panic/Anxiety Disorder<br>2 Borderline Personality Disorder<br>1 Trichotillomania<br>9 Multiple Comorbidities	4 Subclinical Anxiety Symptoms
Psychiatric Treatment during the acute stage	16 Antidepressants<br>3 Mood Stabilisers<br>13 Antipsychotics<br>11 Sedatives	21 Antidepressants<br>3 Mood Stabilisers<br>4 Antipsychotics<br>5 Sedatives	1 Sedatives (*4y prior to participation in the study*)

N.B. Dashes indicate measure was not taken. Abbreviations: AN (Anorexia Nervosa); p-AN (post-acute Anorexia Nervosa); HC (Healthy Control); DASS-21 (21-Item Depression, Anxiety and Stress Scale; Lovibond & Lovibond, 1995); BAQ (Body Awareness Questionnaire; Shields et al., 1989); TAS-20 (20-Item Toronto Alexithymia Scale; Bagby et al., 1994); OCD (Obsessive Compulsive Disorder). Multiple comorbidities include at least two of the following: OCD, Mood Disorder, Borderline Personality Disorder, Panic/Anxiety Disorder. * Heart rate was recorded at the beginning of the session (for 5 minutes) and was then divided by 5 to obtain the average number of heartbeats per minute. [1]Linear regressions were run to examine group differences, with HC as the intercept. As expected, there were significant between group differences on BMI, consistent with the inclusion criteria, but the BMI differences between the p-AN and HC groups were unexpected and thus the effect of BMI was controlled for where necessary. Given some of the expected group differences in psychometric traits, these were taken into account in the exploratory analyses. Bolded values denote statistical significance ($p<0.05$).

Psychometric Measures

To explore whether traits and symptoms associated with AN had any effects on our main, behavioural results, we ran exploratory analyses with questionnaires assessing traits of depression, anxiety, stress (DASS-21; Lovibond & Lovibond, 1995), alexithymia (Bagby et al., 1994), attentiveness to normal, internal bodily processes and sensations (body awareness questionnaire (BAQ); Shields et al., 1989), as well items from an insight into illness questionnaire developed by the research team (see Table S2).

Depression, Anxiety, Stress Scale (DASS-21; Lovibond & Lovibond, 1995): The DASS-21 was only used in a subset of the sample (N_{AN}=27, $N_{p\text{-}AN}$ = 23, N_{HC}=33) due to clinical time constraints. The DASS-21 is a reliable scale (Cronbach's α can range between 0.86 and 0.90; (Gloster et al., 2008) and has 21 items used to assess any symptoms relating to negative mood and emotions on a 4-point Likert scale (0 – *Did not apply to me to all* to 3 – *Applied to me very much*). It has three subscales to assess depression, anxiety, and stress, with higher scores relating to higher levels of depression, anxiety, and stress, with cut-offs being 5, 4, and 8, respectively. The DASS-21 has positive psychometric properties in samples of adults with anxiety and depression. The present sample showed an excellent internal consistency, Cronbach's α= 0.958.

Toronto Alexithymia Scale (TAS-20; Bagby et al., 1994): The TAS-20 was used as a measure of alexithymia as it is the most commonly used self-report questionnaire, with demonstrated good reliability and factorial validity in both clinical and non-clinical populations (González-Arias et al., 2018). All participants, except for two AN and one p-AN participants, completed the TAS-20, which consists of 20 items rated on a 5-point likert scale of 1 (*strongly disagree*) to 5 (*strongly agree*). Total scores may range from 20 to 100 (sum of the three subscale scores), and a total score of 61 or above is generally indicative of alexithymia (Franz et al., 2008; Honkalampi et al., 2001). The total TAS-20 score was used in the present experiment's analysis, instead of each subscale score separately due to the relatively small sample sizes and subsequent power-related issues. In the current experiment, the TAS-20 showed an excellent internal consistency, Cronbach's α= 0.913.

Body Awareness Questionnaire (BAQ; Shields et al., 1989): The BAQ was used to measure self-reported interoceptive sensibility. Due to clinical time constraints the BAQ was only completed by a subset of the sample (N_{AN}=24, $N_{p\text{-}AN}$ =22, N_{HC}=26). The BAQ is an 18-item

scale measuring attentiveness to normal, internal bodily processes and sensations, with good internal consistency as shown both in an undergraduate student sample, Cronbach's α = .82 (Shields et al., 1989) and a clinical sample (Cronbach's α = 0.786; Unal et al., 2021). In this experiment, the BAQ demonstrated a high internal consistency, with Cronbach's α =0.851 in the present sample. The BAQ has four factors: the ability to note responses in body processes, and predict bodily sensations, sleep-wake cycle, and onset of illness. All responses are measured on a 7-point likert scale of 0 (*Not at all true about me*) to 7 (*Very true about me*), with higher scores indicating greater body awareness.

Insight into Illness: Finally, a subset of the AN and p-AN participants completed an insight into illness questionnaire developed by the research team (see Table S2), given that to the best of our knowledge, there is no questionnaire that focusses only on insight in eating disorders. Specifically, there is one self-report measure of insight into illness in eating disorders but not specific to AN only (Konstantakopoulos et al., 2011; Konstantakopoulos, Georgantopoulos, et al., 2020) which was developed largely for clinical, psychiatric use. However, at the time of study design and recruitment it had not yet been validated, its content was too general for the current experiment's scope, and its completion also did not fit within the time limits of the present experiment. Instead, participants from the two clinical groups answered five questions on different dimensions of restrictive, anorexic symptom awareness. The focus was placed on three dimensions, namely on the (i) General, Allocentric Perspective of Self, (ii) Health Consequences, and (iii) Future Perspectives: Hopes and Fears. The self-report items measure patients' awareness of their emaciated appearance and illness, severity, the negative consequences of their insight disordered eating and fears and beliefs regarding behaviours which may hinder prognosis. Each question was rated on a 0 (*not at all*) to 10 (*very much*) likert scale, and each participant's clinician (both for AN and p-AN groups) also rated symptom and illness severity on these questions based on their professional evaluation of the individual's symptoms and the individual's medical files. Individual scores were averaged to obtain a 'subjective' global clinical insight score, with higher scores suggesting more insight into illness, while clinician scores were averaged to obtain an objective score, with higher scores indicating greater illness severity.

Table S2. *Items of the Insight into Illness Questionnaire*

**Item**	**Dimension of insight**

**1**	*How severe is your condition*?	***General, Allocentric Perspective of Self***
**2**	*Do you think that your eating behaviour could have negative consequences for your health?*	***Health Consequences***
**3**	*Do you think that your body weight and shape could have negative consequences for your health?*	
**4**	*Are you afraid of losing control of your eating behaviour?*	***Future Perspectives: Hopes and Fears***
**5**	*Do you believe that the severity of your condition will improve in the future?*	

Data Analysis

Here, we outline the further analyses that were conducted for *Experiment 1*. Specifically, given that we found age and average heart rate group differences, these two measures were added as covariates in our analyses examining group differences on Prior Prospective Self-Efficacy Beliefs. Next, we ran control analyses for Performances within each group separately to control for potential effects of BMI, as well as in each time interval separately to ensure that overall Performance results were not exclusively driven by any one of the intervals. Control analyses with age and average heart rate as predictors were conducted to control for the effects of these factors on Performance, however they did not affect our main findings. Moreover, given that false feedback was randomly given to participants, irrespective of their Performance in the task, three control linear regressions with performance as IV and false feedback as dependent variable (DV) were also carried out. These latter analyses were conducted to look for possible within-group differences in Performance and to control that positive false feedback was not randomly given to participants with higher Performance scores and negative false feedback to participants with lower Performance Scores. Then, we ran linear regressions with Prior Prospective Self-Efficacy Beliefs and Performance as our two separate DVs and group and psychometric measures as interacting independent variables (IVs), as well as with illness duration and severity (in the AN and p-AN groups, separately), as covariates. Finally, in the AN group exploratory analyses were conducted to look at the relationship between their Prior Prospective Self-Efficacy Beliefs and Performance and their clinical insight scores

(calculated as the difference between the clinician- and patient-ratings on each item; Table S2).

Experiment 1: Supplementary Results

Control analyses for Prior Prospective Self-Efficacy Beliefs and Performance

We added age and average heart rate as covariates to examine if they influenced the group effect on Prior Prospective Self-Efficacy Beliefs (Table S3a) and Performance (S3b-f), separately. However, this was not the case. Moreover, BMI was not added as an IV in the linear regression with Performance as the DV given that it was a criterion for the three groups, but three separate regressions were conducted to examine whether BMI affected Performance within each group, since recent criticisms of the heartbeat counting task suggest that BMI may affect sensitivity to heartbeat detection (Richard et al., 2019), and in turn Performance. However, none of the three within-group analyses yielded significant results, suggesting that in the present cohort, Performance was not significantly affected by BMI within each group (Table S3c). Finally, we also found no group differences in Performance (as expected) when looking at each time interval separately (Tables S3d-f).

Table S3. *Control analyses for Prior Prospective Self-Efficacy Beliefs and Performance*

Predictors	β(*SE*)	t	95% CI	*p*
Table S3a. Group Differences on Prior Prospective Self-Efficacy Beliefs. Controlling for Age and Average Heartrate				
Intercept	58.23(*12.60*)	4.62	33.25 - 83.21	*<0.001*
AN	-16.89(*5.54*)	-3.05	-27.88 - -5.91	***0.003***
p-AN	-5.95(*5.49*)	-1.09	-16.83 - 4.92	*0.280*
AGE	0.15(*0.29*)	0.5	-0.44 - 0.73	*0.618*
Average Heartrate	-0.12(*0.14*)	-0.83	-0.40 - 0.16	*0.411*
R^2 / Adj R^2	0.09 / 0.06			
Table S3b. Group Differences on Performance. Controlling for Age and Average Heartrate.				
Intercept	69.41(*12.39*)	5.6	44.86 - 93.97	*<0.001*
AN	-2.17(*5.45*)	-0.4	-12.97 - 8.63	*0.692*
p-AN	5.01(*5.42*)	0.92	-5.73 - 15.76	*0.357*
AGE	0.29(*0.29*)	1.01	-0.28 - 0.87	*0.314*
Average Heartrate	-0.26(*0.14*)	-1.86	-0.54 - 0.02	*0.065*
R^2 / Adj R^2	0.08 / 0.04			
Table S3c. Control Analyses for BMI effects on Performance (within each Group separately)				
Intercept[1]	83.20(*26.21*)	3.18	30.45 – 135.96	*0.002*

BMI	-1.24(*1.65*)	-0.75	-4.56 – 2.08	*0.456*
R^2 / Adj R^2: 0.012 / -0.009; [1]*in AN Group*				
Intercept[2]	61.95(*27.12*)	2.29	7.19 – 116.72	*0.028*
BMI	0.17(*1.36*)	0.12	-2.58 – 2.91	*0.903*
R^2 / Adj R^2: 0.00 / -0.024; [2]*in the p-AN Group*				
Intercept[3]	33.42(*24.32*)	1.37	-15.57 – 82.40	*0.176*
BMI	1.24(1.12)	1.11	-1.01 – 3.49	*0.274*
R^2 / Adj R^2: 0.027 / 0.005; [3]*in the HC Group*				
Table S3d. Group Differences on Performance (25-second trial). Controlling for Age.				
Intercept	55.42(*7.65*)	7.25	40.30 - 70.54	*<0.001*
AN	1.36(*4.69*)	0.29	-7.91 - 10.64	*0.772*
p-AN	3.38(*4.84*)	0.7	-6.19 - 12.94	*0.486*
AGE	0.26(*0.27*)	0.94	-0.28 - 0.79	*0.350*
R^2 / Adj R^2	0.01 / -0.01			
Table S3e. Group Differences on Performance (45-second trial). Controlling for Age.				
Intercept	55.62(*7.62*)	7.3	40.54 - 70.69	*<0.001*
AN	4.48(*4.66*)	0.96	-4.73 - 13.70	*0.338*
p-AN	5.54(*4.83*)	1.15	-4.02 - 15.10	*0.254*
AGE	0.20(*0.27*)	0.75	-0.33 - 0.74	*0.456*
R^2 / Adj R^2	0.02 / -0.006			
Table S3f. Group Differences on Performance (65-second trial). Controlling for Age.				
Intercept	49.01(*7.54*)	6.5	34.09 - 63.93	*<0.001*
AN	1.83(*4.61*)	0.4	-7.28 - 10.94	*0.692*
p-AN	6.61(*4.77*)	1.39	-2.83 - 16.05	*0.168*
AGE	0.34(*0.27*)	1.28	-0.19 - 0.87	*0.204*
R^2 / Adj R^2	0.03 / 0.004			

Abbreviations: AN (Acute Anorexia Nervosa Group); p-AN (post-acute Anorexia Nervosa Group); HC (Healthy Controls Group). Bolded values denote statistical significance (*p<0.05*).

Control analysis for Post False-Feedback Retrospective Self-Efficacy Belief

We ran three linear regressions (one per group), to control for potential effects of false feedback on performance. As expected, no statistically significant effect was found, i.e., the arbitrary false feedback that participants received was not based on their performance in the HCT (Table S4).

Table S4. *Control Analysis for Effect of False Feedback on Performance*

Predictors	β(*SE*)	t	95% CI	*p*
Intercept[1]	61.32(*3.95*)	15.52	53.36 – 69.28	*<0.001*
Positive False Feedback	1.23(*5.16*)	0.24	-9.17 – 11.62	*0.813*

R^2 / Adj R^2: 0.001 / -0.021; [1]in the AN Group				
Intercept [2]	61.14(*4.80*)	12.73	51.41 – 70.87	*<0.001*
Positive False Feedback	4.94(*6.88*)	0.72	-9.00 – 18.88	*0.477*
R^2 / Adj R^2: 0.014 / -0.013; [2]in the p-AN Group				
Intercept[3]	61.45(*5.03*)	12.21	51.28 – 71.62	*<0.001*
Positive False Feedback	-4.46(*7.48*)	-0.60	-61.45 – 34.62	*0.554*
R^2 / Adj R^2: 0.009 / -0.016; [3]in the HC Group				

Abbreviations: AN (Acute Anorexia Nervosa Group); p-AN (post-acute Anorexia Nervosa Group); HC (Healthy Controls Group); PE (Prediction Error). Bolded values denote statistical significance (*p<0.05*).

Analyses with Psychometric Traits and Items from the Insight into Illness Questionnaire

None of the analyses with questionnaire scores (as interacting variables with Group) were significant (Table S5). We also did not find a significant effect illness duration or severity (Table S6), or items from the insight into illness questionnaire on the clinical groups' Prior Prospective Self-Efficacy Beliefs and Performance (Table S7).

Table S5. *Analyses with Psychometric Traits as Interacting Predictors with Group*

Predictors	β(SE)	t	95% CI	*p*
Effect of Depression x Group on Prior Prospective Self-Efficacy Beliefs				
Intercept	62.21(*5.99*)	10.39	50.24 – 74.17	*<0.001*
AN	-19.45(*12.05*)	-1.61	-43.54 – 4.64	*0.112*
p-AN	-0.26(*10.02*)	-1.02	-30.29 – 9.77	*0.31*
Depression	-0.02(*1.45*)	-0.02	-2.92 – 2.87	*0.987*
AN x Depression	0.03(*1.63*)	0.02	-3.24 – 3.29	*0.988*
p-AN x Depression	-0.33(*1.65*)	-0.2	-3.62 – 2.96	*0.842*
R^2 / Adj R^2	0.14 / 0.08			
Effect of Depression x Group on Performance				
Intercept	58.32(*6.33*)	9.22	45.67 – 70.96	*<0.001*
AN	19.91(*12.74*)	1.56	-5.55 – 45.37	*0.123*
p-AN	8.60(*10.59*)	0.81	-12.57 – 29.77	*0.42*
Depression	-1.37(*1.53*)	-0.9	-4.44 – 1.49	*0.373*
AN x Depression	0.34(*1.73*)	0.2	-3.11 – 3.79	*0.845*
p-AN x Depression	1.54(*1.74*)	0.89	-1.94 – 5.02	*0.379*
R^2 / Adj R^2	0.10 / 0.03			
Effect of Anxiety x Group on Prior Prospective Self-Efficacy Beliefs				
Intercept	60.25(*6.37*)	9.46	47.52 – 72.98	*<0.001*

AN	-17.89(*10.04*)	-1.78	-37.96 – 2.19	*0.080*
p-AN	-7.43(*9.79*)	-0.76	-27.00 – 12.13	*0.450*
Anxiety	0.99(*2.34*)	0.43	-3.68 – 5.66	*0.672*
AN x Anxiety	-0.94(*2.51*)	-0.37	-5.95 – 4.07	*0.71*
p-AN x Anxiety	-1.60(*2.51*)	-0.64	-6.63 – 3.42	*0.526*
R^2 / Adj R^2	0.15 / 0.08			
Effect of Anxiety x Group on Performance				
Intercept	59.94(*6.76*)	8.87	46.43 – 73.45	*<0.001*
AN	12.49(*10.66*)	1.17	-8.82 – 33.80	*0.246*
p-AN	9.62(*10.39*)	0.93	-11.15 – 30.39	*0.358*
Anxiety	-2.78(*2.48*)	-1.12	-7.73 – 2.18	*0.267*
AN x Anxiety	1.75(*2.66*)	0.66	-3.57 – 7.07	*0.513*
p-AN x Anxiety	2.58(*2.67*)	0.97	-2.75 – 7.91	*0.337*
R^2 / Adj R^2	0.098 / 0.03			
Effect of Stress x Group on Prior Prospective Self-Efficacy Beliefs				
Intercept	59.83(*8.28*)	7.23	43.29 – 76.38	*<0.001*
AN	-11.77(*14.82*)	-0.79	-41.38 – 17.85	*0.430*
p-AN	-15.43(*12.65*)	-1.22	-40.72 – 9.87	*0.227*
Stress	0.39(*1.17*)	0.33	-1.95 – 2.73	*0.739*
AN x Stress	-0.80(*1.47*)	-0.54	-3.74 – 2.14	*0.590*
p-AN x Stress	0.11	0.07	-2.85 – 3.06	*0.943*
R^2 / Adj R^2	0.12 / 0.08			
Effect of Anxiety x Group on Performance				
Intercept	55.75(8.92)	6.25	37.92 – 73.57	*<0.001*
AN	18.29(*15.96*)	1.15	-13.61 – 50.19	*0.256*
p-AN	12.40(*13.63*)	0.91	-14.85 – 39.65	*0.366*
Stress	-0.19(*1.26*)	-0.15	-2.71 – 2.34	*0.884*
AN x Stress	-0.51(*1.58*)	-0.32	-3.68 – 2.65	*0.747*
p-AN x Stress	0.20(*1.59*)	0.13	-2.98 – 3.39	*0.898*
R^2 / Adj R^2	0.07 / -0.004			
Effect of Body Awareness (BAQ) x Group on Prior Prospective Self-Efficacy Beliefs				
Intercept (HC)	17.92(*24.24*)	0.74	-30.52 – 66.35	*0.463*
AN	3.56(*30.55*)	0.12	-57.49 – 62.62	*0.908*
p-AN	42.26(*31.51*)	1.34	-20.72 – 105.24	*0.185*
BAQ	0.34(*0.29*)	1.18	-0.24 – 0.93	*0.244*
AN x BAQ	-0.14(*0.39*)	-0.36	-0.91 – 0.63	*0.721*
p-AN x BAQ	-0.51(*0.39*)	-1.28	-1.29 – 0.28	*0.204*
R^2 / Adj R^2	0.09 / 0.02			
Effect of Body Awareness (BAQ) x Group on Performance				

Intercept (HC)	55.52(*22.63*)	2.45	10.30 – 100.73	*0.017*
AN	2.07(*28.74*)	0.07	-55.37 – 59.51	*0.943*
p-AN	3.42(*29.98*)	0.11	-56.48 – 63.33	*0.909*
BAQ	0.11(*0.27*)	0.41	-0.43 – 0.66	*0.682*
AN x BAQ	-0.07(*0.36*)	-0.18	-0.79 – 0.66	*0.858*
p-AN x BAQ	-0.05(*0.37*)	-0.14	-0.80 – 0.69	*0.886*
R^2 / Adj R^2	0.01 / -0.07			
Effect of Alexithymia (TAS-20) x Group on Prior Prospective Self-Efficacy Beliefs				
Intercept (HC)	73.73(*14.71*)	5.01	44.64 – 102.82	*<0.001*
AN	-9.86(*18.74*)	-0.53	-46.93 – 27.21	*0.600*
p-AN	-11.74(*17.74*)	-0.66	-46.83 – 23.35	*0.509*
TAS-20	-0.53(*0.38*)	-1.41	-1.27 – 0.21	*0.162*
AN x TAS-20	0.14(*0.42*)	0.33	-0.69 – 0.96	*0.743*
p-AN x TAS	0.25(*0.42*)	0.59	-0.59 – 1.09	*0.558*
R^2 / Adj R^2	0.13 / 0.09			
Effect of Alexithymia (TAS-20) x Group on Performance				
Intercept (HC	81.23(*14.38*)	5.65	52.78 – 109.68	*<0.001*
AN	-8.01(*18.46*)	-0.43	-44.52 – 28.50	*0.665*
p-AN	-3.51(*17.48*)	-0.2	-38.08 – 31.07	*0.841*
TAS-20	-0.55(*0.37*)	-1.5	-1.27 – 0.17	*0.135*
AN x TAS-20	0.38(*0.41*)	0.939	-0.42 – 1.19	*0.349*
p-AN x TAS-20	0.30(*0.41*)	0.733	-0.52 – 1.12	*0.465*
R^2 / Adj R^2	0.05 / 0.01			

N.B. Depression, Anxiety, and Stress were measured using the DASS-21 Scale; body awareness was measured using the BAQ (Body Awareness Questionnaire; Shields et al., 1989); alexithymia was measured using the TAS-20 (20-Item Toronto Alexithymia Scale; Bagby et al., 1994). Abbreviations: AN (Acute Anorexia Nervosa Group); p-AN (post-acute Anorexia Nervosa Group); HC (Healthy Controls Group). Bolded values denote statistical significance ($p<0.05$).

Table S6. *Effects of Illness Duration and Severity on Prior Prospective Self-Efficacy Beliefs and Performance*

Predictors	β(*SE*)	t	95% CI	*p*
Effect of Illness Duration on Prior Prospective Self-Efficacy Beliefs (in the AN group only)				
Intercept	36.58(*4.60*)	7.95	27.32 – 45.84	*<0.001*
Illness Duration	0.23(*0.37*)	0.629	-0.51 – 0.98	*0.532*
R^2 / Adj R^2	0.008 / -0.013			
Effect of Illness Duration on Performance (in the AN group only)				
Intercept	61.77(*351*)	17.59	54.71 – 68.84	*<0.001*
Illness Duration	0.12(*0.28*)	0.45	-0.43 – 0.68	*0.656*

R^2 / Adj R^2	0.004 / -0.02			
Effect of Illness Severity on Prior Prospective Self-Efficacy Beliefs (in the AN group only)				
Intercept	26.56(*7.75*)	3.43	54.71 – 68.84	*0.001*
Illness Severity	0.25(*0.13*)	1.95	-0.43 – 0.68	*0.058*
R^2 / Adj R^2	0.08 / 0.06			
Effect of Illness Severity on Performance (in the AN group only)				
Intercept	62.94(*5.92*)	10.62	51.02 – 74.86	*<0.001*
Illness Severity	0.01(*0.10*)	0.14	-0.18 – 0.21	*0.888*
R^2 / Adj R^2	0.00 / -0.02			
Effect of Illness Duration on Prior Prospective Self-Efficacy Beliefs (in the p-AN group only)				
Intercept	41.64(*5.24*)	7.95	31.03 – 52.25	*<0.001*
Illness Duration	1.38(*0.88*)	1.57	-0.41 – 3.16	*0.126*
R^2 / Adj R^2	0.06 / 0.04			
Effect of Illness Duration on Performance (in the p-AN group only)				
Intercept	65.57(*5.24*)	12.52	54.94 – 76.19	*<0.001*
Illness Duration	0.31(*0.88*)	0.36	-1.47 – 2.10	*0.724*
R^2 / Adj R^2	0.004 / -0.024			
Effect of Illness Severity on Prior Prospective Self-Efficacy Beliefs (in the p-AN group only)				
Intercept	44.89(*7.81*)	7.45	29.05 – 60.74	*<0.001*
Illness Severity	0.05(*0.11*)	0.46	-0.17 – 0.27	*0.650*
R^2 / Adj R^2	0.006 / -0.022			
Effect of Illness Severity on Performance (in the p-AN group only)				
Intercept	70.27(*8.07*)	8.70	53.87 – 86.66	*<0.001*
Illness Severity	-0.03(*0.11*)	-0.30	-0.26 – 0.19	*0.763*
R^2 / Adj R^2	0.003 / -0.03			
Abbreviations: AN (Acute Anorexia Nervosa Group); p-AN (post-acute Anorexia Nervosa Group). Bolded values denote statistical significance (*p<0.05*).				

Table S7. *Effects of Insight into Illness on Prior Prospective Self-Efficacy Beliefs and Performance (AN Group only)*

Predictors	β(*SE*)	t	95% CI	*p*
Effect of Illness Severity Error on Prior Prospective Self-Efficacy Beliefs				
Intercept	52.52(*8.00*)	6.57	36.05 – 68.99	*<0.001*
Severity Error Score	-3.37(*2.25*)	-1.5	-8.01 – 1.27	*0.147*
R^2 / Adj R^2	0.08 / 0.05			
Effect of Illness Severity Error on Performance				
Intercept	0.63(*0.06*)	10.18	0.50 – 0.76	*<0.001*
Severity Error Score	0.01(*0.02*)	0.35	-0.03 – 0.04	*0.733*
R^2 / Adj R^2	0.005 / -0.035			
Effect of Health Consequences Error Score on Prior Prospective Self-Efficacy Beliefs				

Intercept	42.96(*5.51*)	7.71	31.12 – 53.80	*<0.001*
Health Consequences	0.26(*2.10*)	0.12	-4.08 – 4.59	*0.903*
R^2 / Adj R^2	0.001 / -0.039			
Effect of Health Consequences Error Score on Performance				
Intercept	0.68(*0.04*)	17.86	0.61 – 0.76	*<0.001*
Health Consequences	-0.03(*0.01*)	-1.92	-0.06 – 0.00	*0.066*
R^2 / Adj R^2	0.13 / 0.09			
Effect of Future Perspectives on Prior Prospective Self-Efficacy Beliefs				
Intercept	57.57(*16.90*)	3.41	22.76 – 92.37	*0.002*
Fear	-1.84(*2.02*)	-0.01	-5.99 – 2.31	*0.370*
R^2 / Adj R^2	0.03 / ‘-0.006			
Effect of Future Perspectives on Performance				
Intercept	0.82(*0.12*)	6.67	0.57 – 1.07	*<0.001*
Fear	-0.02(*0.01*)	-1.45	-0.05 – 0.01	*0.160*
R^2 / Adj R^2	0.08 / 0.04			

N.B. For illness severity and health consequences an error score was computed by subtracting patients’ scores from the clinician’s respective score on that item. Error scores could range from -10 to +10, with a score of -10 indicating negative bias (i.e., patients see their condition and the effects as more severe than the clinician), a score of 0 indicating no bias between patient and clinician perspective, and a score of +10 indicating positive bias (i.e., patients see their condition and the effects as less severe than the clinician). In the future perspectives the average of the two (fear of losing control over eating behaviour in the future and hopes for illness severity improving in the future) items was used (patient scores only used here, and the rating on the fear item (item number 5, see Table 2.2 in Chapter 2 was reverse scored). For the ‘Health Consequences’ dimension an average of the two error scores from the two items in that dimension was calculated.

Supplementary Methods: Experiment 2

Participants

Experiment 2 had a non-overlapping sample to *Experiment 1* but with the same eligibility criteria and with identical recruitment sites. However, for *Experiment 2* we added an additional recruitment site – AN and p-AN participants were also recruited at the collaborating National and Kapodistrian University of Athens (NKUA) 1st and 2nd psychiatric clinics (Greece) and HCs were also recruited from the University of Athens (Greece). Although total of N_{AN}=40, $N_{p\text{-}AN}$ =40, and N_{HC}=121 were screened, N_{AN}=4 were excluded due to other comorbid Eating Disorders; N_{HC}=1 was excluded as upon screening they said they were on psychiatric medication; and, N_{HC}=1 was excluded due to having a BMI >35 (significantly higher than our BMI upper limit of 25; see Table S8 for participant clinical characteristics). The rest of the screened participants were recruited and tested, however N_{AN}=1 wished to withdraw halfway during the session; and N_{HC}=2 were excluded from all analyses due to a technical failure during the HCT and failure to comply with task instructions, respectively. Institutional and local ethics approval was granted and written informed consent was obtained from all participants prior to their participation. HCs from the UK site received either monetary compensation at a fixed rate of £7.5/hour, or university course credits. The participants from the Italian site received £10 Amazon vouchers. The participants from the Greek site were not allowed to receive compensation in line with the Ethics regulations from the local institutions. The OSF project including the preregistration of *Experiment 2*, raw data and R code can be accessed at: https://osf.io/x4ysv. Data were analysed using R (R, Boston, MA) and figures were generated using ggplot (Wickham, 2016).

Table S8. *Experiment 2* Participant Clinical Characteristics

	AN (N=35)	p-AN (N=40)	HC (N=117)
Psychiatric Comorbidities	2 Depression	2 Depression	2 Subclinical Depression Symptoms
	1 GAD	2 GAD	6 Subclinical Anxiety Symptoms
	3 OCD	1 OCD	
	4 Personality Disorder	6 Personality Disorder	
	1 Learning Disability		
Current Medication	4 Antidepressants	6 Antidepressants	

5 Sedatives	2 Sedatives
4 Antipsychotics	7 Antipsychotics
5 SSRIs	7 SSRIs
1 Anxiolytics	3 Anxiolytics

N.B. Anxiety disorders includes panic disorders. Dashes indicate measure not taken. Abbreviations: AN (Anorexia Nervosa); p-AN (post-acute AN); HC (Healthy Control Group); GAD (Generalised Anxiety Disorder); OCD (Obsessive-Compulsive Disorder); SSRIs (Selective Serotonin Reuptake Inhibitors).

Psychometric Measures

To examine whether traits and symptoms associated with AN had any effects on our main, behavioural results, we ran exploratory analyses with questionnaires assessing eating behaviours and responses to emotional states (EDI-3; Garner, 2004), obsessive and compulsive symptoms (Foa et al., 2002), reactions to uncertainty, ambiguous situations and the future (IUS-12; Carleton et al., 2007), depression, anxiety, stress (Lovibond & Lovibond, 1995), alexithymia (Bagby et al., 1994), and insight into dimensions of restrictive, anorexic symptoms.

Eating Disorder Inventory (EDI-3; Garner, 2004): The EDI-3 is a self-report measure used to assess disordered eating behaviour (e.g., bulimia and the tendency to think about and engage in binge-eating), as well as other psychological behaviours that are highly relevant, but not necessarily specific, to eating disorders. The EDI-3 is widely used in interoception research given that certain of its items assess response to emotional states, confusion related to affective and bodily functioning and is also used to assess the presence of an eating disorder. The 12-subscale questionnaire consists of 91 items rated on a 6-point Likert scale, ranging from 0 (*Always*) to 4 (*Rarely*; *Always* and *Usually* both receive a score of 0). We used the Interoceptive Deficits subscale (EDI-3-ID) which is consisted of 9 items as a precision proxy. The internal consistency of the total EDI-3 score and the EDI-3-ID subscale in our sample was excellent with Cronbach’s α scores of 0.96 and 0.95, respectively.

Obsessive-Compulsive Inventory (OCI-R; Foa et al., 2002): The OCI-R is an 18-item self-rating scale typically used to assess the severity and type of obsessive-compulsive symptoms. The OCI-R evaluates six groups of OCD symptoms on a 5-point Likert scale ranging from 0 (*not at all*) to 4 (*extremely*), with three items in each group and a total score obtained by

summing all items and a total score range of 0-72. Higher scores indicate greater OCD symptoms. A cut-off score of 21 has been introduced, with scores equal to or higher than 21 indicating the possible presence of OCD symptoms. Overall, the OCI-R has shown great overall internal consistency and good test-retest reliability (OCI-R; Foa et al., 1998; Wootton et al., 2015). The discriminant validity of the OCI-R remains unclear; namely, the ability of the OCI-R to distinguish OCD from other types of anxiety (e.g., worry and general anxiety) is not fully examined. The internal consistency in our sample was excellent, with Cronbach's α=0.91.

Intolerance of Uncertainty (IUS-12; Carleton et al., 2007): The IUS-12 questionnaire was used to assess reactions to uncertainty, ambiguous situations and the future. Items are scored on a 5-point Likert scale ranging from 1 (*Not at all characteristic of me*) to 5 (*Entirely characteristic of me*), with possible total scores ranging from 27 to 135, and higher scores suggest greater intolerance of uncertainty. The IUS-12 consists of two subscales, the prospective anxiety subscale (PAS) and the inhibitory anxiety subscale (IAS) and has good internal consistency. Finally, the IUS-12 is significantly correlated with state measures of worry (Penn State Worry Questionnaire; Meyer et al., 1990; Van Rijsoort et al., 1999) and has a lower correlation with measures of trait anxiety (trait version of the State-Trait Anxiety Inventory; Spielberger et al., 1971; Van der Ploeg, 1980). Internal consistency in our sample was excellent, with Cronbach's α of 0.94.

Depression, Anxiety, Stress Scale (DASS-21; Lovibond & Lovibond, 1995): The DASS-21 was used as a scale to assess symptoms relating to negative mood and emotions on a 4-point likert scale ranging from 0 (*did not apply to me at all*) to 3 (*applied to me very much*; see details above). The internal consistency in our sample was excellent with Cronbach's α =0.95.

Toronto Alexithymia Scale (TAS-20; Bagby et al., 1994): The TAS-20 was used to measure alexithymia (see details above). In the present experiment we used the total score in our analysis, and not each subscale scores in separate analyses due to the relatively small sample sizes and subsequent power-related issues. In the current experiment, the TAS-20 showed an excellent internal consistency, Cronbach's α=0.87.

Insight into Illness: The AN and p-AN groups completed a 12-item questionnaire on explicit beliefs about illness and symptomatology developed by the research team (Table S9, which was an updated version of the questionnaire used in *Experiment 1*, Table S2). The questionnaire measures four dimensions of restrictive, anorexic symptom awareness, namely the (i) General, Allocentric Perspective of Self, (ii) Health Consequences, (iii) Perspective Taking, and (iv) Future Perspectives: Hopes and Fears (hereafter referred to as Counterfactual beliefs). The self-report items measure patients' awareness of their emaciated appearance and illness severity, the negative consequences of their disordered eating, their beliefs regarding others' perspectives on their conditions, and finally, their fears and beliefs regarding behaviours which may hinder prognosis. Each question is rated on a 0 (*not at all*) to 10 (*very much*) scale, and each participant's clinician (both for AN and p-AN groups) also rates symptom and illness severity on these questions based on their professional evaluation of the individual's symptoms and the individual's medical files. To obtain insight scores we calculated an error score using the clinician-score weighted difference between patient and clinician scores, with a negative score suggesting deficits into insight. We obtained an error score for each of the 8 insight questionnaire items, and a score per dimension: (i) General, Allocentric Perspective of Self, (ii), Health Consequences, (iii) Perspective Taking).

Table S9. *Insight into Illness Questionnaire*

	**Item**	**Subscale**
1	How severe is your condition? (*Eating disorder*)	***General, Allocentric Perspective of Self***
2	How pathological do you think your approach to food is?	
3	Do you think that you are too thin in comparison to the average woman in the country?	
4	How pathological do you think your eating behaviour is?	
5	Do you think that your eating behaviour could have negative consequences for your health?	***Health Consequences***
6	Do you think that your body weight and shape could have negative consequences for your health?	
7	Do you think that people are correct when they say that you are too thin?	

8	Do you think that people are correct when they say that you do not eat enough?	***Perspective Taking***
9	Are you afraid of losing control of your eating behaviour? *	***Future Perspectives: Hopes and Fears***
10	Do you believe that the severity of your condition will improve in the future?	
11	Are you afraid of gaining weight? *	
12	Do you believe that your approach towards food could improve in the future?	

N.B. All items were scored on a scale of 0 (*not at all*) to 10 (*extremely*). * Denotes that items were reverse scored. Each patient's clinician was given items 1 to 8 and asked to answer based on their clinical evaluation of the patient's state and the respective clinical file. Patient scores on items 9-12 were used in separate analyses on everyday, counterfactual beliefs and their relation to prospective beliefs about interoception.

Secondary Hypotheses, Measures and Analyses

In a secondary set of hypotheses and analyses, we were interested in examining how our three groups differed in their beliefs on their ability prior to completing the HCT. To do this we compared our groups' Prior Prospective Self-Efficacy Beliefs, expecting that the beliefs of the AN and p-AN groups would be more 'pessimistic' about their abilities to perform well in the HCT as indexed by their lower values as compared to the HCs, as in *Experiment 1*. We examined this by running a MLM to assess whether Group predicted differences in Prior Prospective Self-Efficacy Beliefs, while controlling for Age, and adding Study Site as a random effect. Although we found no significant group differences on Prior Prospective Self-Efficacy Beliefs (see below), we explored whether traits and behaviours often encountered in populations with AN explained the pattern of our results, in order to also better understand the prospective pessimistic beliefs of the clinical populations (i.e., Posterior Prospective Self-Efficacy Beliefs).

After participants completed the HCT, they also provided a Posterior Retrospective Self-Efficacy Belief, where they were asked to assess how well they were able to feel their heartbeats. We examined whether there would be group differences in Performance estimation retrospectively, expecting that the two clinical groups misestimated their Performance in the HCT more than HCs as indexed by the greater discrepancy between their

Posterior Retrospective Self-Efficacy Beliefs relative to their Performance. To do this, we calculated a Performance Error Percentage score by multiplying by 100 the Performance-weighted difference between Posterior Retrospective Self-Efficacy Beliefs and Performance. To evaluate between-group differences we used as outcome variable the logarithm of the Performance Error Percentage (although non-preregistered initially, values were logarithmically transformed because the outcome variable followed a lognormal distribution). Performance Error Percentage data were not normally distributed and upon evaluation, the discrepancy between participants' beliefs and their actual performance was better distributed (Figures S4-S6). Hence, although not initially preregistered we decided to also examine the difference between Beliefs and Performance (outcome variable) between our three groups (predictor variable) and within each group, separately.

Then, we were interested in evaluating whether our two clinical groups underestimated Performance in the cardiac interoception task, and specifically whether they would show poorer interoceptive awareness (correspondence between Performance and Performance Confidence Estimates) in comparison to HCs. We predicted that the two clinical groups would show poorer interoceptive awareness in comparison to HCs. We examined these differences by running Pearson's correlations between participants' Performance and Performance Confidence – correlations were run across groups, and within each group separately. However, these analyses could not indicate the extent to which bottom-up interoceptive signals (e.g., cardiac signals during HCT) with regards to top-down prior beliefs, and interoceptive traits influence the precision of metacognitive, prospective beliefs (here, Posterior Prospective Self-Efficacy Belief). Hence, to elucidate to what extent the discrepancy between Performance and interoceptive sensibility (subjective measure of one's ability to attend to interoceptive signals; here, measured via the interoceptive deficits subscale form the EDI-3; (EDI-3-ID; Garner, 2004) explain interoceptive awareness, we obtained an interoceptive trait prediction error (ITPE) z-score (here, calculated as the difference between the Performance z-score and the EDI-3-ID z-score; see Garfinkel et al., 2016 for details). We predicted that the AN and p-AN groups would underestimate their Performance in the cardiac interoception task in comparison to HCs as indexed by their more negative ITPE values. The ITPE score was then converted into a z-score, with a positive value suggesting that participants overestimated their interoceptive abilities, whilst a negative one suggested an underestimation of one's own interoceptive abilities. Then, we ran a linear regression to examine whether group predicted ITPE z-score differences.

After each belief (e.g., Prior Prospective Self-Efficacy Belief) and heartbeat counting trial, participants were asked to report how confident they were in the accuracy of their answer. We then examined whether our groups differed in confidence throughout different experimental stages, and also used the confidence estimates after each heartbeat counting trial (Performance Confidence) in our aforementioned Bayesian Belief Updating and interoceptive awareness analyses. Given findings of low confidence on the HCT in a previously reported AN sample (Kinnaird et al., 2020) we expected that the clinical groups would be less confident in the accuracy of their answers throughout the experiment, especially during the HCT task and in their Posterior Retrospective Self-Efficacy Beliefs. To examine this, we ran the same analyses as we did for Prior and Posterior Prospective Self-Efficacy Beliefs for Prior Prospective, Performance, and Posterior Prospective Confidence to assess between-group differences.

Then, we ran a series of control analyses on our primary measures. Given criticisms on the effects of time awareness in HCT performance (Brener & Ring, 2016), we added a control task during which participants, for the same time intervals, estimated how much time they thought had passed, rather than how many heartbeats they felt. Additional control measures included a 5-min baseline measurement of heart rate variability (HRV; calculated as the root mean square (RMSSD) of the difference in the time elapsed between two consecutive inter-beat intervals), and beliefs about typical heartbeats of oneself and of the general population were also sampled (by asking them to report how many heartbeats they think they have/the general population has per minute when at rest), for the same control purposes based on previous literature showing that individual differences in both time estimation and knowledge about heartbeats may influence performance on heartbeat counting (Brener & Ring, 2016; Knapp-Kline & Kline, 2005). We then ran an analysis using Performance as our outcome variable, Group as the predictor variable, and Time Accuracy, knowledge about heartbeats in the general population and in the self as control variables. Time accuracy was calculated using the same formula we used for Performance (1) and replacing actual recorded heartbeats with the duration of the respective time trial (in sec) and the estimated, felt heartbeats with participants' estimation of the time elapsed (in sec). The Self-Heartbeat Accuracy score was calculated as the difference between the samples' average heartbeat and the participant's response regarding their own average heart rate when at rest, weighted by the sample average. The General-Heartbeat Accuracy score was calculated in the same way but replacing

the sample average heartbeat with the general population average heartbeat scores when at rest (80) and using participants' response on the average heart rate of the population when at rest, instead of their own. We also ran an analysis on group differences in HRV but given the lack of significant findings HRV was not added in any further analyses (see below). We then ran additional, control analyses for Performance in each time interval separately to ensure that overall Performance results were not exclusively driven by any one of the intervals. We also carried out preliminary analyses to see if BMI was different between groups, as BMI was a group-categorisation criterion. As expected, BMI was significantly lower in the AN vs HC group, but we did not find a statistically significant difference between the p-AN and HC groups. Given criticisms on the effects of BMI on interoceptive abilities (Richard et al., 2019), we ran within group tests to examine potential effects of BMI on Performance (i.e., interoceptive accuracy).

Furthermore, to exclude the possibility that difficulties in updating prospective self-efficacy beliefs relate to a domain-general cognitive inflexibility, we also tested cognitive flexibility and particularly set-shifting using the Wisconsin Card Sorting Task (WCST; Grant & Berg, 1948) in a subset of our *Experiment 2* participants (AN=11; p-AN =20; HC=34). Moreover, to account for potential effects of cognitive inflexibility on learning rates and beliefs, we first ran between-group differences on the percentage of correct WCST scores, and then ran a MLM with learning rate (the one that used Performance Confidence as a precision proxy) as our outcome variable, Group as our predictor variable and the WCST as our random effect. We repeated this analysis using participants' percentage of preservative responses; that is, arranging the cards on the basis of former trial criteria (see Arbel et al., 2013).

Additionally, we ran various exploratory analyses to examine the effect of clinical measures and traits on our main variables. To test the validity of our experimental Posterior Prospective Self-Efficacy Beliefs about interoception, in the clinical populations only (both AN and p-AN groups), we also assessed their relation to prospective, everyday beliefs about patients' AN symptoms. Specifically, we explored how everyday fears and hopes (e.g., "*Do you believe that the severity of your condition will improve in the future?*"; "*Are you fearful of gaining weight?*") from a questionnaire developed by the present study's authors, predicted (Prior and Posterior) Prospective Self-Efficacy Beliefs in the AN and p-AN groups (separately). Our first set of analyses used subscale scores (hopes and fears, separately) as standalone IVs, and in the second set of analyses we used the total score (average of the four items from the hopes

and fears subscales). We used patient scores only on future hopes and fears as our predictor variables and self-efficacy beliefs as our outcome variables. Given that these future hopes and fears items are similar to some EDI-3 items (Garner, 2004), we also ran exploratory correlations between standalone item scores of the EDI-3 and Posterior Prospective Self-Efficacy Beliefs (only in the two clinical groups, separately: Table S21).

Finally, deficits into insight have often been reported in the AN population; we used items from an insight into illness scale developed by the authors (Table S9) of the current study to explore correlations between aspects of insight and self-efficacy beliefs, Performance and learning rates in the AN group only. We report how we scored the items, how we analysed the scores, and the results in more detail below.

Supplementary Results: Experiment 2

Here we present the output from our main analyses, as well as the output of some key secondary measures (namely Prior and Retrospective Self-Efficacy Beliefs, and Interoceptive Awareness).

1.1 Main Measures (Performance and Posterior Prospective Self-Efficacy Beliefs) – Control and Exploratory Analyses

Additional analyses to examine whether Performance was influenced by participant ID and study site, BMI, time awareness, knowledge about heartbeats and heart-rate variability (HRV) are presented in Tables S10 and S11

We ran a multiple linear regression with Performance as our outcome variable, Group, Time Accuracy, and Self- and General-Heartbeat Accuracy scores as our predictor variables. However, we found no significant effects (Table S10a). We then examined whether Performance differed in each time interval separately but found no significant differences (Table S10b). Next, BMI was not added as an IV in the main MLM for between-group difference on Performance given that it was a criterion for grouping participants (as expected the AN group had a significantly lower BMI than the HCs; Table S10c), but we conducted three separate regressions to examine whether BMI affected Performance within each group, separately. None of the three within-group analyses yielded significant results, suggesting that in our cohort, Performance was not significantly affected by BMI (Table S10d). Finally, we found no between-group HRV differences (Table S10e), thus HRV was not added as a control variable in our Performance analysis.

Table S10. *Additional and Control Analyses for Performance*

S10a. Performance (IAcc) with participant ID and Study Site as Random Effects				
Predictors	β(*SE*)	*t*	95% CI	*p*
(Intercept)	56.18(*5.77*)	*9.74*	44.78 – 67.59	*<0.001*
AN	-0.89(*6.98*)	*-0.13*	-14.68 – 12.91	*0.899*
p-AN	-2.48(*6.08*)	*-0.41*	-14.49 – 9.53	*0.684*
Time Accuracy	-0.13(*0.07*)	*-1.92*	-0.26 – 0.00	*0.057*
Self-Heartbeat Accuracy	-20.15(*14.45*)	*-1.40*	-48.72 – 8.41	*0.156*
General-Heartbeat Accuracy	-5.99(*12.18*)	*-0.49*	-30.08 – 18.10	*0.624*
S10b. Group Differences in Performance Per Trial Duration				
(Intercept)[1]	0.40(*0.03*)	*13.41*	0.34 – 0.46	*<0.001*
AN	0.10(*0.06*)	*1.59*	-0.02 – 0.22	*0.113*
p-AN	-0.02(*0.06*)	*-0.40*	-0.14 – 0.10	*0.693*
[1]*This analysis is for the 25sec long trial*				
(Intercept)[2]	0.37(*0.03*)	*11.49*	0.30 – 0.43	*<0.001*
AN	-0.09(*0.09*)	*-0.92*	-0.27 – 0.10	*0.360*
p-AN	0.10(*0.07*)	*1.35*	-0.05 – 0.24	*0.181*
[2]*This analysis is for the 30sec long trial*				
(Intercept)[3]	0.40(*0.03*)	*14.38*	0.35 – 0.46	*<0.001*
AN	0.07(*0.06*)	*1.17*	-0.05 – 0.18	*0.245*
p-AN	0.03(*0.06*)	*0.47*	-0.09 – 0.14	*0.641*
[3]*This analysis is for the 45sec long trial*				
(Intercept)[4]	0.41(*0.03*)	*14.59*	0.35 – 0.46	*<0.001*
AN	0.06(*0.06*)	*1.12*	-0.05 – 0.18	*0.264*
p-AN	-0.004(*0.06*)	*-0.06*	-0.12 – 0.11	*0.950*
[4]*This analysis is for the 65sec long trial*				
S10c. BMI Differences Between Groups				
HC (Intercept)	20.48(*0.53*)	*38.30*	19.41 - 21.54	*<0.001*
AN	-4.35(*0.93*)	*-4.69*	-6.19 - -2.51	***<.001***
p-AN	-0.54(*1.05*)	*-0.52*	-2.62 - 1.53	*0.604*
S10d. Performance (IAcc) within each group				
AN	15.13(*60.53*)	*0.25*	-110.41 – 140.66	*0.805*
BMI	2.32(*3.74*)	*0.62*	-5.43 – 10.06	*0.542*
p-AN	64.82(*78.53*)	*0.83*	-104.84 - 234.48	*0.424*
BMI	-1.18(*3.93*)	*-0.3*	-9.67 - 7.32	*0.77*

HC	42.58(*18.86*)	*2.26*	4.61 – 80.56	*0.029*
BMI	0.26(*0.90*)	*0.28*	-1.55 - 2.06	*0.777*
S10e. Heart Rate Variability (HRV) Differences Between Groups				
HC (Intercept)	5.92(*0.27*)	*21.83*	5.39 - 6.46	*<0.001*
AN	0.72(*0.72*)	*1.01*	-0.70 - 2.14	*0.316*
p-AN	0.07(*0.62*)	*-0.12*	-1.30 - 1.16	*0.909*

N.B. For Self-Heartbeat Knowledge, participants were asked to report how many heartbeats they think they have per minute. We then obtained a self-accuracy score per participant, by weighing the difference between the participant's actual heartbeats per minute and their answer with the number of heartbeats the participant had in a minute when at rest. For General-Heartbeat Knowledge participants were asked to report how many heartbeats they think the average person has per minute when at rest. A score on General-Heartbeat accuracy was obtained by weighing the difference between the actual heartbeat average in the general population and the participant's answer with the average in the general population. Given average heartbeats per minute in the general population may range from 60 to 100 beats per minute (bpm; Sapra et al., 2023) we used the average (i.e., 80bpm). Bolded values denote statistical significance ($p<0.05$). Participant ID refers to each participant's response per trial.

Additional analyses to explore what drives the lower Posterior Prospective Self-Efficacy Beliefs of the AN group.

We wanted to further examine the potentially mediating role of traits and symptoms seen in AN and their effect on Posterior Prospective Self-Efficacy Beliefs, using the Baron and Kenny (1986) steps for mediation analysis. A mediation analysis can be performed after a significant effect of the IV has been found on the DV. In this case, Group (IV) significantly predicted differences in Posterior Prospective Self-Efficacy Beliefs (DV). Hence, we proceeded to completing the three mediation analysis steps. These involve (i) establishing a significant effect of the IV on the mediating variable (MV) of interest, (ii) establishing a significant effect of the MV on the DV, and (iii) adding both the IV and MV in a regression model to examine if there is a partial, full, or no mediation effect. Here, given that we had multiple variables of interest, we first ran a correlation between the Depression, Anxiety, and Stress subscale scores from DASS-21, participants' IUS-12, OCI-R, TAS-20, and EDI-3-ID scores. Given the high correlation among all those measures, we first ran a principal component analysis (PCA) and used the 7 resulting principal components in 2 separate linear regressions to examine their effect on self-efficacy beliefs. We had to run two separate linear regressions (one with the all 7 principal components and one with the IUS-12, OCI-R, TAS-20, and EDI-3-ID components only) given that participants from one of our sites did not complete the DASS-21 questionnaire. To further explore what drives the lower Posterior

Prospective Self-Efficacy Beliefs of the clinical groups (in comparison to the HCs'), we ran an MLM using the principal component scores as predictors and Study Side as a random effect. We found that Depression and Stress scores as measured using the DASS-21 questionnaire (Lovibond & Lovibond, 1995), but not the other psychometric traits had a significant effect on Posterior Self-Efficacy Beliefs (Table S11a). We complemented this analysis by running non-preregistered linear regressions on Posterior Self-Efficacy Beliefs, to explore whether Depression and Stress would explain the group effects previously observed. Indeed, we found a significant effect in both analyses, suggesting that part of the observed pessimism in self-efficacy beliefs could be attributed to depression and stress. To further examine whether this was a state or trait effect we ran Holm-corrected linear regressions within each clinical group separately, and using the Depression and Stress principal component scores as our two separate predictors we found a significant effect of Depression on the p-AN group's Posterior Prospective Self-Efficacy Beliefs, but not on the AN group's, and no significant effect of Stress on neither group's Posterior Prospective Self-Efficacy Beliefs. To better understand the pattern, we plotted the data and saw that as Depression and Stress scores increase, Posterior Self-Efficacy Beliefs tend to decrease (Figures S1 and S2). However, after a certain threshold of Depression and Stress, the Posterior Prospective Self-Efficacy Beliefs plateau.

We then ran a non-preregistered, exploratory analyses to explore whether set-shifting difficulties explained the difference on our groups' Posterior Prospective Self-Efficacy Beliefs. However, as seen on Table S11b, we found no significant effects of the WCST percentage of correct and preservative scores on participants' Posterior Prospective Self-Efficacy Beliefs. We discuss these findings and their implications in detail in the Discussion.

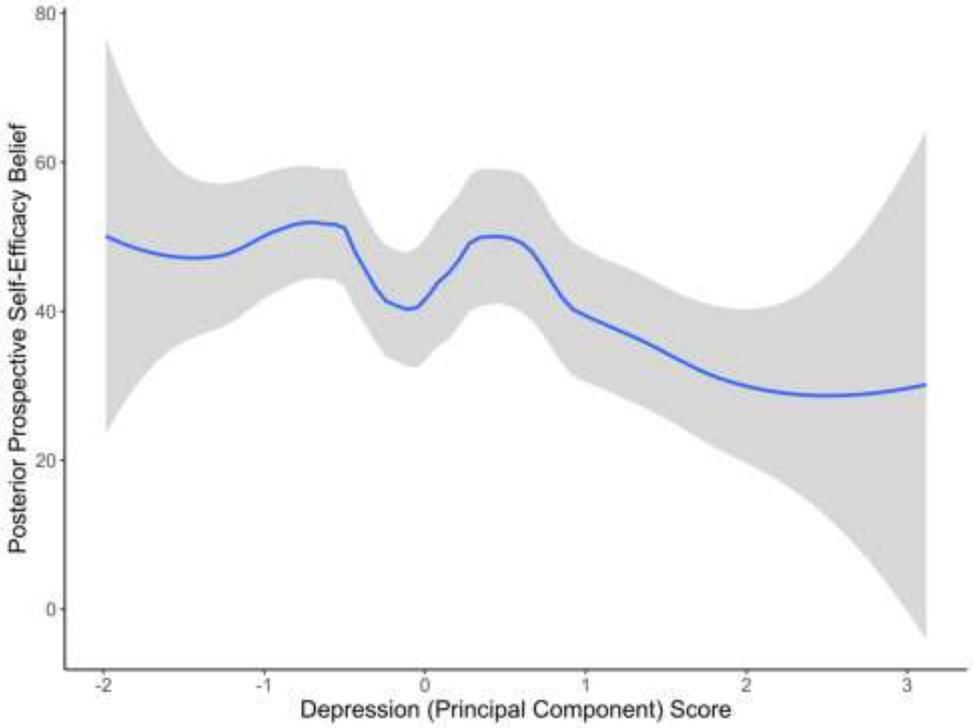

Figure S1. Visual representation of the Posterior Prospective Self-Efficacy Beliefs decreasing as the Depression Scores increase, and after a certain threshold (2), the Self-Efficacy Belief values reach a plateau.

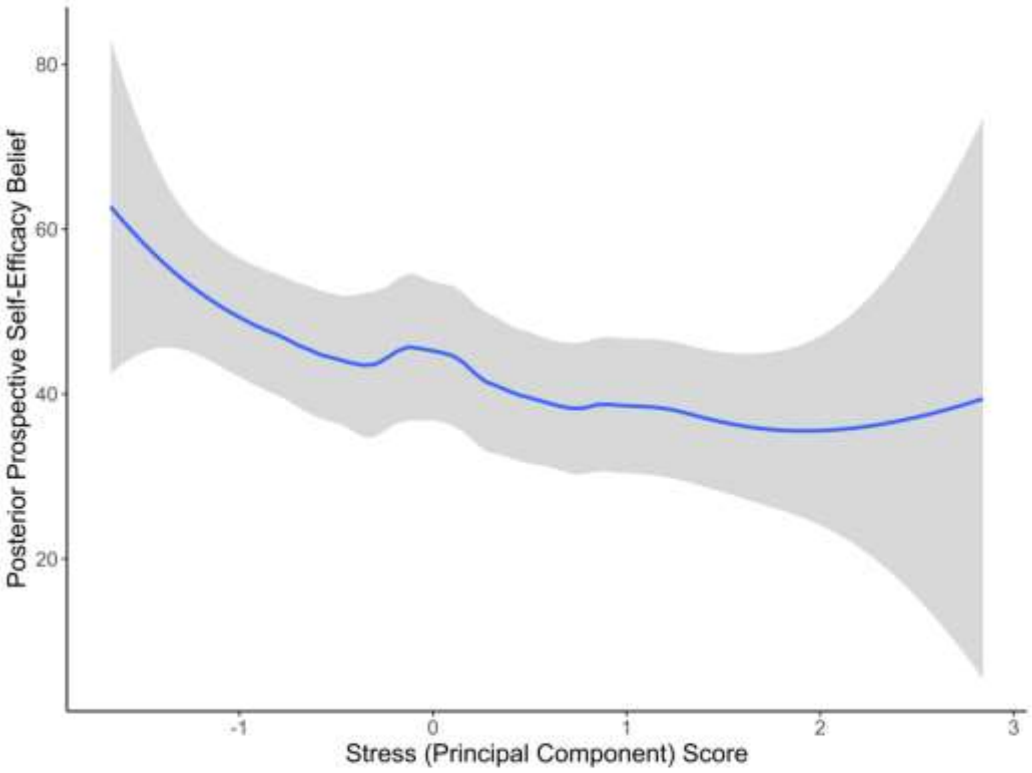

Figure S2. Visual representation of the Posterior Prospective Self-Efficacy Beliefs decreasing as the Stress Scores increase, and after a certain threshold (1), the Self-Efficacy Belief values reach a plateau.

**Table S11.** *Additional Analyses on Posterior Prospective Self-Efficacy Beliefs*				
S11a: Analysis Using the Depression, Stress, Anxiety, IUS-12, OCI-R, TAS-20 and EDI-3-ID Principal Components as Predictors				
Predictors	β(*SE*)	*t*	95% CI	*p*
(Intercept)	43.93(*1.93*)	*22.74*	40.15 – 47.72	*<0.001*
Depression	-4.96(*1.89*)	*-2.63*	-8.66 – -1.26	***0.009***

Stress	-5.64(*1.92*)	*-2.94*	-9.40 – -1.88	***0.003***
Anxiety	0.77(*1.90*)	*0.40*	-2.96 – 4.50	*0.687*
IUS-12	0.97(*1.80*)	*0.54*	-2.55 – 4.49	*0.589*
OCI-R	-1.02(*1.91*)	*-0.54*	-4.77 – 2.72	*0.592*
TAS-20	2.04(*1.95*)	*1.05*	-1.77 – 5.86	*0.294*
EDI-3-ID	2.07(*2.10*)	*0.98*	-2.06 – 6.19	*0.326*
**Random Effects**				
σ^2	431.35			
τ_{00} STUDY SITE	0.00			
N STUDY SITE	3			
Observations	120			
Marginal R^2 / Conditional R^2	0.127/NA			
Analysis Using the Depression Principal Component as the Predictor				
(Intercept)	45.24(*2.37*)	*19.08*	40.55 – 49.94	*<0.001*
AN	-1.79(*5.34*)	*-0.33*	-12.37 – 8.79	*0.739*
p-AN	-5.88(*5.98*)	*-0.98*	-17.73 – 5.97	*0.328*
Depression	-4.77(*2.08*)	*-2.29*	-8.89 – -0.64	***0.024***
Holm-corrected linear regressions in each clinical group separately, using the Depression Principal Component as the Predictor				
(Intercept: AN)	40.61(*5.32*)	*7.63*	29.54 – 51.68	*<0.001*
Depression	-0.81(*4.01*)	*-0.20*	-9.15 – 7.53	*0.841*
(Intercept: p-AN)	39.97(*6.39*)	*6.26*	26.16 – 53.77	*<0.001*
Depression	-14.49(*5.31*)	*-2.73*	-25.95 - -3.02	***0.034***
Analysis Using the Stress Principal Component as the Predictor				
(Intercept)	45.04(*2.37*)	18.99	40.35 – 49.74	*<0.001*
AN	-3.16(*5.13*)	-0.62	-13.32 – 7.00	*0.539*
p-AN	-3.03(*6.16*)	-0.49	-15.23 – 9.17	*0.624*
Stress	-5.01(*2.04*)	-2.46	-9.05 – -0.97	***0.016***
Holm-corrected linear regressions in each clinical group separately, using the Stress Principal Component as the Predictor				
(Intercept: AN)	41.00(*4.79*)	8.55	31.03 – 50.97	*<0.001*
Stress	-2.63(*4.84*)	-0.54	-12.70 – 7.44	*0.593*
(Intercept: p-AN)	47.93(*8.78*)	5.46	28.95 – 66.90	*<0.001*
Stress	-15.05(*8.56*)	-1.76	-33.54 – 3.44	*0.204*
Analysis Using the IUS-12, OCI-R, TAS-20 and EDI-3-ID Principal Components				
(Intercept)	45.90(*2.19*)	20.93	41.60 – 50.20	*<0.001*
IUS-12	-1.04(*1.66*)	-0.63	-4.30 – 2.22	*0.532*

OCI-R	-2.23(*1.68*)	-1.32	-5.53 – 1.07	*0.186*
TAS-20	-0.93(*1.68*)	-0.55	-4.22 – 2.36	*0.580*
EDI-3-ID	-1.36(*1.75*)	-0.78	-4.78 – 2.06	*0.436*
**Random Effects**				
σ^2	509.33			
τ_{00} STUDY SITE	7.28			
ICC	0.01			
N STUDY SITE	4			
Observations	187			
Marginal R^2 / Conditional R^2	0.017/0.031			
S11b: Analyses on Posterior Prospective Self-Efficacy Beliefs with the WCST Scores				
Analysis with Group and WCST Percentage Score as the Predictor Variables				
(Intercept)	49.06(*11.77*)	4.17	25.69 – 72.43	*<0.001*
AN	-16.36(*7.54*)	-2.17	-31.32 - -1.40	***0.032***
p-AN	-7.80(*6.00*)	-1.30	-19.71 – 4.11	*0.197*
WCST_PCT	0.04(*0.016*)	0.26	-0.27 – 0.35	*0.799*
Analysis with Group and WCST Preservative Percentage Score as the Predictor Variables				
Intercept	49.02(*7.45*)	6.58	34.22 – 63.82	*<0.001*
AN	-16.66(*7.69*)	-2.17	-31.93 - -1.39	***0.033***
p-AN	-7.92(*6.22*)	-1.28	-20.30 – 4.42	*0.205*
WCST_PRES_PCT	0.09(*0.23*)	0.40	-0.37 – 0.55	*0.693*

N.B. We used the principal component values. Abbreviations: IUS-12 (Intolerance of Uncertainty Scale); OCI-R (Obsessive Compulsive Inventory Revised); TAS-20 (20-Item Toronto Alexithymia Scale); EDI-3-ID (Interoceptive Deficits Subscale from the Eating Disorders Inventory 3). Depression, Stress and Anxiety refer to the subscale scores from the DASS-21 (21-Item Depression, Anxiety and Stress Scale); WCST_PCT (Percentage of Correct WCST Score); WCST_PRES_PCT (Percentage of WCST Preservative Score). Bolded values denote statistical significance ($p < 0.05$).

1.2 Secondary Measures Analysis Results

Women at the acute and post-acute state of AN did not provide significantly poorer Prior Prospective Self-Efficacy Beliefs compared to HCs.

Although we predicted women with acute and post-acute AN would provide poorer Prior Prospective Self-Efficacy Beliefs on average compared to HCs, a linear regression showed that group was not a statistically significant predictor of participants' Prospective Self-Efficacy Beliefs prior to completing the HCT (Figure S3; Table S12).

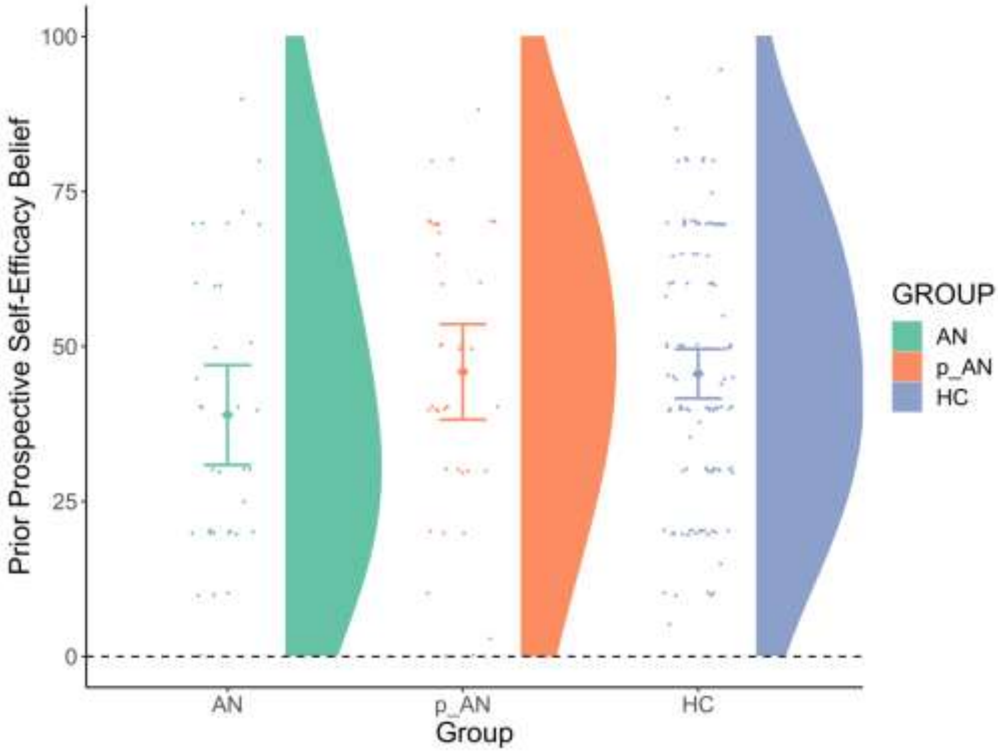

Figure S3. Average of Prior Prospective Self-Efficacy Beliefs per group

Table S12. *Analysis for Group Differences on Prior Prospective Self-Efficacy Beliefs, with Age as Control Variable and Study Site as Random Effect*

Predictors	β(*SE*)	*t*	*95% CI*	*p*
(Intercept)	41.68(*6.91*)	*6.91*	28.14 – 55.23	*<0.001*
AN	-6.24(*4.37*)	*4.37*	-14.81 – 2.32	*0.153*
p-AN	0.01(*4.21*)	*4.21*	-8.23 – 8.26	*0.997*
AGE	0.16(*0.27*)	*0.27*	-0.36 – 0.68	*0.552*
**Random Effects**				
σ^2	499.96			
τ_{00} STUDY SITE	0.00			
N STUDY SITE	4			
Observations	189			
Marginal R^2 / Conditional R^2	0.015 / NA			

Performance Error Percentage and Difference

In the preregistration, we reported we were going to analyse the performance error percentage differences (misestimation of performance) between our groups. As seen on the histogram below (Figure S4), our data were positively skewed to the right, following a log-normal distribution. Hence, we logarithmically transformed the values, and the distribution became more normal as seen on the histogram below (Figure S5). Although not initially

preregistered, we also ran a between-group analysis on the difference between participants' retrospective beliefs and actual performance (IAcc), given that the values were more normally distributed (Figure S6).

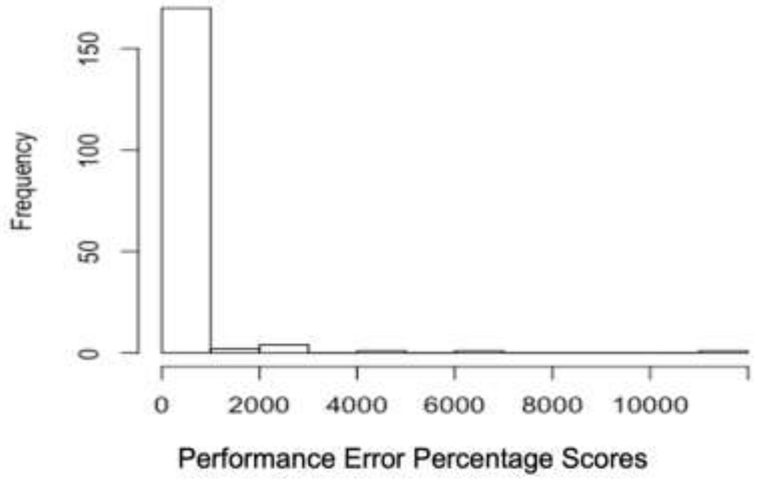

Figure S4. Histogram of Performance Error Percentage Scores before being logarithmically transformed.

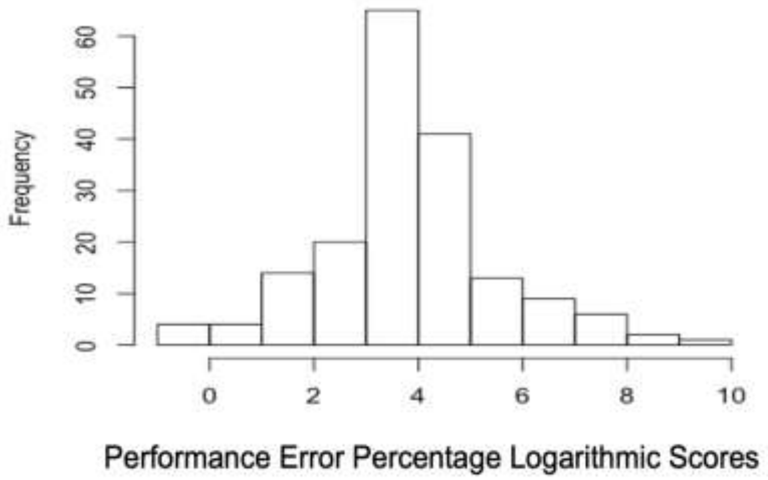

Figure S5. Histogram of the Logarithmically transformed Performance Error Percentage Scores.

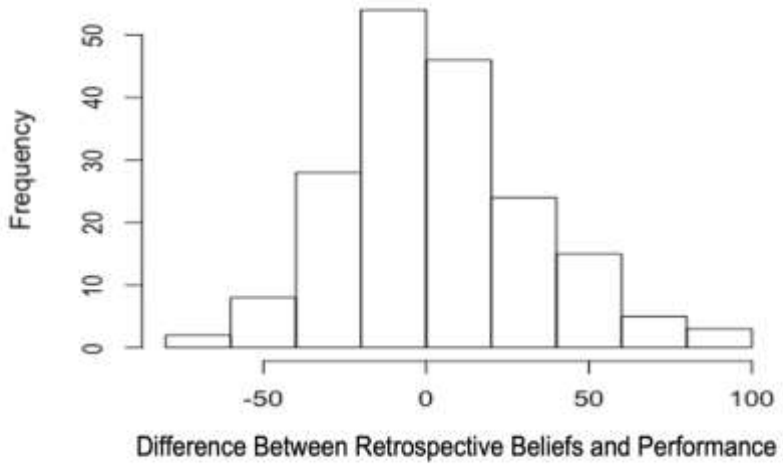

Figure S6. Histogram of the difference between Posterior Retrospective Self-Efficacy Beliefs and Performance.

Women with acute and post-acute AN underestimated their Performance Retrospectively, while HCs overestimated their Performance

We expected that the clinical groups would significantly misestimate their performance retrospectively, as indexed by their greater Performance Error Percentage Scores in comparison to the HCs. However, we found non-significant differences between participants' logarithmically transformed Performance Error Percentage Scores (Table S13a). We also examined the difference between Posterior Retrospective Self-Efficacy Beliefs and Performance (non-preregistered) and found this misestimation to differ in the two clinical groups relative to the HCs (Table S13b). Specifically, the difference between beliefs and performance on the task was positive for the HCs and negative for both clinical groups. Further within-group comparisons examining the main effects revealed that the HCs significantly overestimated their Performance (i.e., they predicted they would do better than they did in reality), while the two clinical groups underestimated their Performance (although this effect was not statistically significant; Table S13c). In summary, even though there is a difference in misestimation between the groups, we cannot claim that the clinical groups misestimate more or less than HCs, due to the opposite directionality of these differences.

Table S13. *Output of secondary, behavioural analyses for Experiment 2*

S13a: Performance Error Percentage (Log)				
Predictors	β(*SE*)	*t*	95% CI	*p*
HC (Intercept)	3.43(*0.54*)	*6.32*	2.36 – 4.50	*<0.001*
AN	0.02(*0.34*)	*0.06*	-0.65 – 0.69	*0.950*
p-AN	0.07(*0.34*)	*0.21*	-0.60 – 0.74	*0.837*
AGE	0.02(*0.02*)	*0.75*	-0.03 – 0.06	*0.453*
S13b: Difference between Posterior Retrospective Self-Efficacy Beliefs and Performance				
HC (Intercept)	-1.82(*9.13*)	*-0.20*	-19.82 – 16.19	*0.842*
AN	-15.44(*5.77*)	*-2.68*	-26.82 – -4.07	***0.008***
p-AN	-13.99(*5.67*)	*-2.45*	-25.18 – -2.81	***0.015***
AGE	0.43(*0.35*)	*1.23*	-0.26 – 1.13	*0.221*
S13c: Difference between Posterior Retrospective Self-Efficacy Beliefs and Performance within Group				

AN	-7.73(*4.87*)	*-1.59*	-17.62 - 2.17	*-0.122*
p-AN	-4.33(*5.19*)	*-0.83*	-14.87 - 6.22	*0.410*
HC	8.86(*2.73*)	*3.24*	3.45 - 14.27	***0.002***

N.B. Bolded values denote statistical significance ($p < 0.05$).

Women with AN showed a correlation between their Performance and Performance Confidence Estimates

To evaluate whether our two clinical groups underestimated Performance in the cardiac interoception task, and specifically whether they would show poorer interoceptive awareness (correspondence between Performance and Performance Confidence Estimates), we ran Pearson's correlations, across groups and also within each group separately. There was a non-significant (r=-0.066, p=.375) correlation between Performance and Performance Confidence across all groups. When examining correlations between Performance and Performance confidence within each group separately we found a significant relationship in women with AN, such that higher Performance Confidence Estimates were related to higher Performance (r_{AN}=.439, p_{AN}=.008). The correlation between these two variables was not significant in the p-AN and HC groups ($r_{p\text{-}AN}$=-.240, $p_{p\text{-}AN}$ =.158; r_{HC}=-.144, p_{HC}=.127). Contrary to our prediction, these findings suggests that the AN group may have better Interoceptive Awareness compared to the p-AN and HC groups.

Women with AN showed a poorer correlation between their Interoceptive Sensibility scores and Interoceptive Accuracy (i.e., Performance) than HCs

To better understand the extent to which the discrepancy between interoceptive sensibility (subjective measure of one's ability to attend to interoceptive signals; here, measured via the interoceptive deficits subscale form the EDI-3; Garner, 2004) and Performance explain interoceptive awareness, we obtained an Interoceptive Trait Prediction Error (ITPE) z-score per participant, expecting the AN and p-AN groups to show an underestimation of their Performance as indexed by their ore negative ITPE z-score values in comparison to HCs.

When looking at the ITPE z-scores at face value (M(SD)), both clinical groups had a negative value ($ITPE_{AN}$=-0.46(1.22), $ITPE_{p\text{-}AN}$ =-0.25(1.61)) suggesting they underestimated their own interoceptive abilities. The HCs had a positive value ($ITPE_{HC}$=0.21(1.56)), suggesting they overestimated their interoceptive abilities. Using a linear regression to examine ITPE z-score differences between groups, we found significant group differences, suggesting that the AN

group significantly underestimated their interoceptive abilities in comparison to HCs, as indexed by the significantly more negative ITPE z-score, yet this was not the case for the p-AN group (Table S14). Our results suggest that in general, the AN group underestimated their Performance, despite the positive correlation we found above, between Performance Confidence and Performance. It is noteworthy, that these analyses do not allow examination of latent variables that may be driving behaviour and thus our focus is placed on the belief updating analyses instead.

Table S14: Output of secondary, behavioural analyses for *Experiment 2*: ITPE z-score Differences Between Groups

Predictors	β(*SE*)	t	95% CI	*p*
HC (Intercept)	0.21(*0.14*)	1.14	-0.07 – 0.49	*0.146*
AN	-0.67(*0.30*)	-2.27	-1.25 – -0.09	***0.025***
p-AN	-0.46(*0.29*)	-1.57	-1.04 – 0.12	*0.118*

N.B. Bolded values denote statistical significance (*p<0.05*).

Women with AN reported significantly lower confidence than HCs following HCT Performance

We predicted that both clinical groups would report significantly lower confidence throughout the experiment in comparison to HCs, especially during the HCT (hereafter referred to as Performance Confidence). We found no significant difference in the Confidence estimates of the p-AN group as opposed to the HCs following Prior and Posterior Prospective Self-Efficacy Beliefs (Table S15a and S15c). In line with our prediction, we found that the AN group reported to be significantly less confident after the HCT trials, i.e., when reporting how confident they were in the number of heartbeats they felt, as opposed to the HCs (Table S15b). We also found a trend towards significance in Posterior Prospective Confidence estimates, in that the AN group were less confident than the HCs (Table S15c).

Table S15. *Analyses with Confidence Estimates between Groups*

S15a: Prior Prospective Confidence

Predictors	β(*SE*)	*t*	95% CI	*p*
HC (Intercept)	67.09(*2.26*)	*29.72*	62.63 - 71.54	*<0.001*
AN	-3.09(*4.69*)	*-0.66*	-12.34 - 6.16	*0.511*

p-AN	-6.09(*4.54*)	*-1.34*	-15.05 - 2.88	*0.182*
S8b: Performance Confidence				
HC (Intercept)	59.08(*2.30*)	*25.64*	54.54 - 63.63	*<0.001*
AN	-17.63(*4.79*)	*-3.68*	-27.07 - -8.18	***<0.001***
p-AN	-6.38(*4.64*)	*-1.37*	-15.53 - 2.78	*0.171*
S8c: Posterior Prospective Confidence Estimate				
HC (Intercept)	72.66(*1.86*)	*39.01*	68.98 - 76.33	*<0.001*
AN	-7.10(*3.87*)	*-1.84*	-14.73 - 0.53	*0.068*
p-AN	-2.94(*3.75*)	*-0.79*	-10.34 - 4.45	*0.433*
N.B. Bolded values denote statistical significance (*p<0.05*).				

1.3 Computational Modelling: Model Presentation and Comparisons, Control and Exploratory Analyses

Computational Modelling Analyses

In the next part of this Results section, we present the target models (Table S16), the baseline model comparison, the analyses ran to examine whether the winning model was better than the competing models, further analyses for participants' precision-weighted Learning Rate, exploratory analyses on between-group differences on the actual Learning Rates, and the winning model validation and description.

Table S16. *Main Target Models*

Model from Table 3	Prior (μ_θ)	Evidence (y)	Precision of the prior (π_θ)	Precision of evidence (π_ε)
1.1., 2.1, 3.1, 4.1	Prior Prospective Self Efficacy Belief	Performance	Prior Prospective Confidence	Performance Confidence
1.2, 2.2., 3.2, 4.2	Prior Prospective Self Efficacy Belief	Posterior Retrospective Self-Efficacy	Prior Prospective Confidence	Performance Confidence
1.3, 2.3, 3.3, 4.3	Prior Prospective Self Efficacy Belief	Performance	Prior Prospective Confidence	EDI-3-ID
1.4, 2.4, 3.4, 4.4	Prior Prospective Self Efficacy Belief	Posterior Retrospective Self-Efficacy	Prior Prospective Confidence	EDI-3-ID

1. Baseline Model Comparison

For validation and control purposes of our main models of interest, we created two sets of baseline models which we then compared. The first set of models (one model per evidence measure) assumed a perfect learning rate (λ =1, the participant uses the evidence as their posterior beliefs). Our second set of baseline models (actually, one model given its baseline nature) assumed no learning (λ = 0, the participant uses the prior as their posterior beliefs). If λ = 0, the models would yield the same score, assuming the same values are used as priors, posteriors, and respective proxies for precision. In the first step for our modelling analyses, we compared our baseline models. To do this, we compared the models using Performance and Posterior Retrospective Self-Efficacy Beliefs as evidence (separate models), and Performance Confidence and EDI-3-ID as different proxies for precision. Due to the high correlation (r=0.87, p<.001) between Posterior Retrospective Self-Efficacy Beliefs (when used as evidence), and Posterior Prospective Self-Efficacy Beliefs, the precision-weighted Learning Rate was very close to 1 (i.e., a perfect learning rate, where the participant uses the evidence as their posterior beliefs). Therefore, our baseline model which assumed 100% learning was the best fit, both across all groups and within each group separately (Models S1.3, S2.3, S3.3, S4.3 in Table S17).

Table S17. *MAE and BIC values for the baseline models*

**Model Comparison across all Participants**	**N**	**MAE**	**BIC**
S1.1 Baseline: 0 Learning	183	14.4	1586
S1.2 Baseline: 100% Learning: Evidence was Performance	183	22.86	1650
S1.3 Baseline: 100% Learning: Evidence was Posterior Retrospective Self-Efficacy	183	7.46	1417
**Model Comparison in the HCs only**			
S2.1 Baseline: 0 Learning	114	13.97	978.8
S2.2 Baseline: 100% Learning: Evidence was Performance	114	23.2	1017
S2.3 Baseline: 100% Learning: Evidence was Posterior Retrospective Self-Efficacy	114	7.19	854
**Model Comparison in the AN group only**			
S3.1 Baseline: 0 Learning	34	16.3	302.2
S3.2 Baseline: 100% Learning: Evidence was Performance	34	19.53	300.7
S3.3 Baseline: 100% Learning: Evidence was Posterior Retrospective Self-Efficacy	34	8.08	268.7
**Model Comparison in the p-AN group only**			
S4.1 Baseline: 0 Learning	35	13.97	312
S4.2 Baseline: 100% Learning: Evidence was Performance	35	25	335.3

S4.3 Baseline: 100% Learning: Evidence was Posterior Retrospective Self-Efficacy	35	7.74	299.4

N.B. MAE refers to Mean Absolute Error and BIC refers to Bayesian Information Criterion. These two measures are used to examine model fit, with smaller values suggesting better model fit.

2. Linear regressions ran to complement the results presented in Figure 4

For Predictions A and B we ran two further, non-preregistered steps. First, after completing our preregistered model comparison for Prediction A, we noticed that the parameter choice in the precision proxy of the evidence made only marginal improvement in the model fit, and the parameter choice in evidence delivered a relatively bigger improvement. Therefore, in a non-preregistered analysis we examined if this improvement in the choice of evidence parameter was statistically significant, while using as a precision proxy of evidence the parameter of the winning model. To accomplish this, we generated the absolute error of each prediction (predicted versus actual Posterior Prospective Self-Efficacy Beliefs) for these two models and compared the two using a linear regression to assess if the predicted absolute errors were significantly smaller in the winning model. Specifically, we examined whether the difference between the Predicted Posterior Self-Efficacy Beliefs using Performance and Posterior Retrospective Self-Efficacy Beliefs as Evidence in the two separate models, and Performance Confidence as the Precision Proxy was statistically significant, i.e., the winning model was significantly better than the other competing models. In the first instance the analysis was ran across all groups, and in the second it was only run in the HC group. Indeed, the model that used Posterior Retrospective Self-Efficacy Beliefs as evidence was significantly better than that using Performance as evidence (both across all participants, and within the HCs; Table S18a).

After completing our preregistered model comparison for Prediction B to identify our winning evidence precision proxy, in non-preregistered analyses we then explored (both across groups and within the HCs only) whether the difference between the two precision proxies (when using Posterior Retrospective Self-Efficacy Beliefs as evidence) was significant. Specifically, we examined whether the difference between the Precision Proxies was statistically significant when using Posterior Retrospective Self-Efficacy Beliefs as the evidence. These analyses were run to explore the statistical significance of the results presented in Figure 4, and to test whether the precision-proxy of the winning model was significantly better than the other proxy used in the competing model (i.e., same evidence measure, but different precision proxy). However, our model comparisons showed that

although EDI-3-ID was a better precision proxy of evidence, it was not significantly better than its competing precision proxy (Performance Confidence; Table S18b). This suggests that irrespective of which precision proxy for the evidence we examine, Posterior Retrospective Self-Efficacy Beliefs as evidence best predict our groups' Posterior Prospective Self-Efficacy Beliefs.

Table S18. *Additional linear regressions for the Belief Updating Predictions.*

S18a: Exploratory analyses for Prediction A				
Predictors	β(*SE*)	*t*	95% CI	*p*
Predicted Posteriors; Difference between Evidences, Performance Confidence as Precision Proxy. Across Groups				
Intercept	5.38(*0.86*)	*6.27*	3.69 - 7.07	***<0.001***
Predicted Posteriors; Difference between Evidences, Performance Confidence as Precision Proxy. Within HCs				
Intercept	5.64(*1.14*)	*4.94*	3.38 - 7.90	***<0.001***
S18b: Exploratory analyses for Prediction B				
Predicted Posteriors; Difference between Precision Proxies, Posterior Retrospective Self-Efficacy Beliefs as Evidence; Across Groups				
Intercept	0.48(*0.29*)	*1.65*	-0.09 – 1.06	*0.100*
Predicted Posteriors; Difference between Precision Proxies, Posterior Retrospective Self-Efficacy Beliefs as Evidence; Within HCs				
Intercept	0.26(*0.25*)	*1.01*	-0.25 – 0.76	*0.315*
N.B. Bolded values denote statistical significance ($p < 0.05$).				

3. Control analyses on precision-weighted Learning Rates with Wisconsin Card Sorting Task (WCST) scores

We ran non-preregistered exploratory analyses using the percentage of correct WCST scores and the percentage of WCST preservative scores to examine whether differences in precision-weighted Learning Rates (when using Performance Confidence as a precision proxy of the evidence) between the groups were explained by set-shifting difficulties. However, neither analysis was significant (Table S19). Therefore, general cognitive flexibility and set-shifting abilities did not influence our patients' learning rates, or their optimality.

Table S19. *Control analyses for Learning Rate using WCST scores*

Analysis with Group and WCST Percentage Score as Predictor Variables				
Predictors	β(*SE*)	*t*	95% CI	*p*
(Intercept)	0.42(*0.08*)	*5.13*	0.26 – 0.58	*<0.001*
AN	-0.07(*0.05*)	*-1.29*	-0.17 – 0.04	*0.199*
p-AN	0.01(*0.04*)	*0.27*	-0.07 – 0.09	*0.789*
WCST_PCT	0.001(*0.001*)	*0.95*	-0.001 – 0.003	*0.334*
Analysis with Group and WCST Preservative Percentage Score as Predictor Variables				
(Intercept)	0.54(*0.05*)	*10.49*	0.44 – 0.64	*<0.001*
AN	-0.06(*0.05*)	*-1.21*	-0.17 – 0.04	*0.229*
p-AN	0.02(*0.04*)	*0.40*	-0.07 – 0.10	*0.688*
WCST_PRES_PCT	-0.002(*0.002*)	*-1.01*	-0.005 – 0.002	*0.316*
N.B. Abbreviations: WCST_PCT (Percentage of Correct WCST Score); WCST_PRES_PCT (Percentage of WCST Preservative Score). Bolded values denote statistical significance.				

4. Analyses on Actual Learning Rates

We explored the actual Learning Rates of our three groups following the assumptions of a Bayesian Learning Framework and analysing our data in the form of a linear regression based on the structural equivalence between the two (see Saramandi et al., 2022 for detailed methodology). Specifically, we ran one linear regression, using in both as our outcome variable participants' Belief Update (i.e., the difference between the Posterior and Prior Prospective Self-Efficacy Beliefs) and as predictor variables the interaction between Group and Prediction Error (calculated as the difference between Posterior Retrospective and Prior Prospective Self-Efficacy Beliefs). In this linear regression, the coefficient of the interaction term corresponds (due to the aforementioned structural equivalence) to the difference between the Learning Rates of our groups.

Interestingly, when comparing participants' actual Learning Rates (we calculated one Learning Rate per group), we found that the p-AN group had a significantly lower actual Learning Rate vs HCs, as predicted, but this difference was not significant when looking at the AN group vs the HCs (Table S20).

Table S20. *Actual Learning Rat Differences Between Groups*

Predictors	β(*SE*)	*t*	*95% CI*	*p*
Intercept	0.08(*0.83*)	*0.10*	-1.56 – 1.72	*0.924*
Prediction Error	0.79(*0.05*)	*16.08*	0.69 – 0.89	***<0.001***

AN x Prediction Error	0.11(*0.11*)	*1.03*	-0.10 – 0.33	*0.305*
p-AN x Prediction Error	-0.21(*0.10*)	*-2.15*	-0.40 – -0.02	***0.033***

N.B. The first linear regression model uses participants' actual Performance as evidence, and the second linear regression model uses participants' Posterior Retrospective Self-Efficacy Beliefs as evidence. Prior refers to Prior Prospective Self-Efficacy Beliefs. Bolded values denote statistical significance.

5. Winning model description and validation

In this section, we describe and assess the equations, parameters and predictions of the model which best predicted participants Posterior Prospective Self-Efficacy Beliefs (also referred to as winning model). All parameters in this model were directly calculated from collected experimental or psychometric measures, and therefore there was no specific methodology for parameter recovery. Overall, the parameters used in the winning model had the expected distributions (with one exception) and produced reliable predictions in line with the actual posterior beliefs.

Model Description:

The models used in this paper were based on the following equation:

$$\mu_{\theta|y} = \mu_\theta + \frac{\pi_\varepsilon}{\pi_\theta + \pi_\varepsilon}(y - \mu_\theta),$$

Where, for our winning model:

$\mu_{\theta|y}$: Posterior Prospective Self-Efficacy Beliefs,

μ_θ : Prior Prospective Self-Efficacy Beliefs,

y : Posterior Retrospective Self-Efficacy Beliefs,

π_θ : Confidence in Prior Prospective Self-Efficacy Beliefs (proxy for precision of prior),

π_ε : EDI-3-ID (interoceptive deficits (using the interoceptive deficits subscale from the Eating Disorders Inventory; Garner, 2004; proxy for precision of evidence)

Additionally, the precision-weighted learning rates (λ) were calculated as follows:

$$\lambda = \frac{\pi_\varepsilon}{\pi_\theta + \pi_\varepsilon}$$

Parameters Validity:

In the histograms below (Figure S7) we present the distributions of the parameters used in the winning model. They do not present any obvious irregularities, with the exception of the precision proxy of the evidence which shows a negative skew. This is not a concern, given that these are actual measurements and not modelled values.

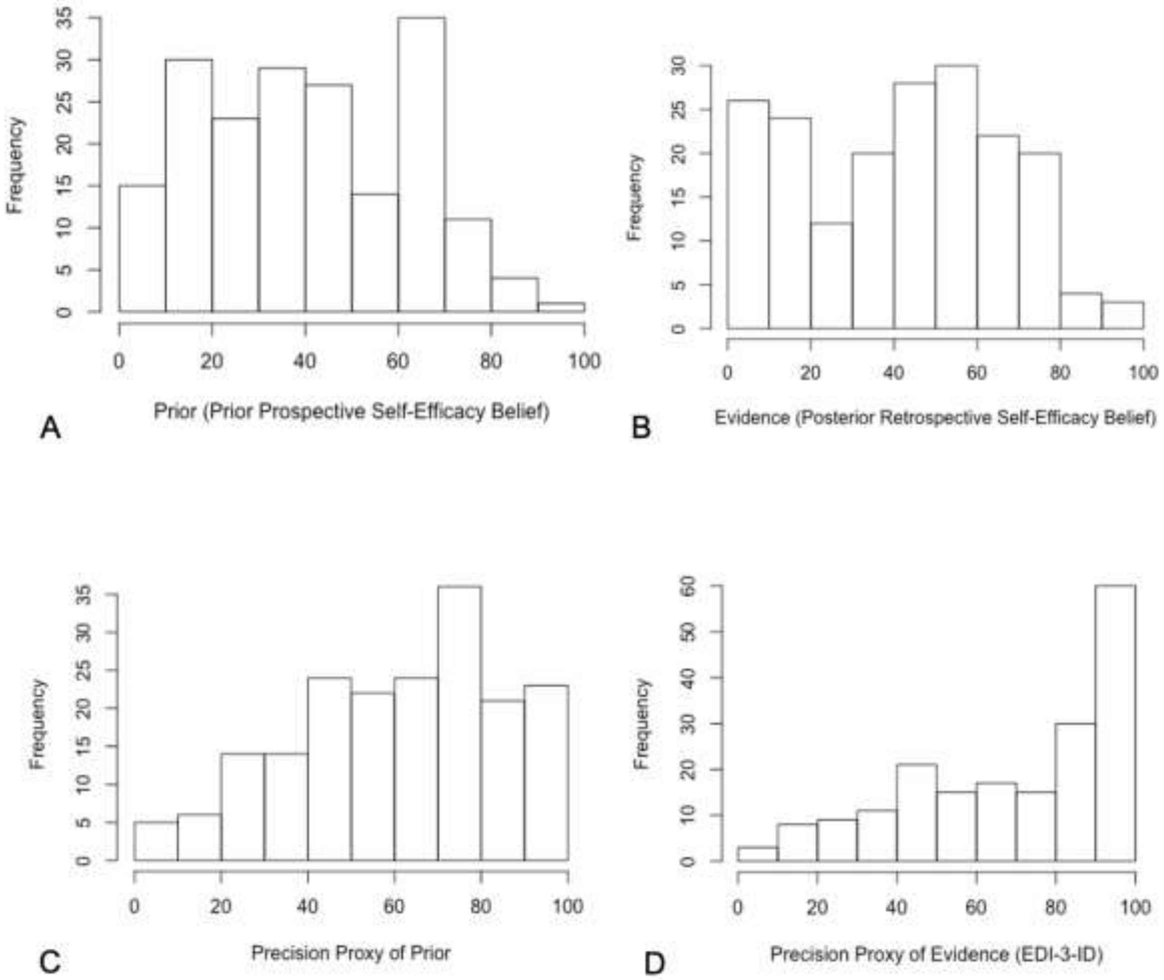

Figure S7: Panel A shows the distribution of the Prior. Panel B shows the distribution of the Evidence. Panel C shows the distribution of the Precision Proxy of the Prior. Panel D shows the distribution of the Precision Proxy of the Evidence of the winning model (when using the EDI-3-ID scores).

<u>Learning Rate:</u>

The learning rate calculated with the winning model's precision proxy of evidence did not present any irregularities, as shown in the histogram below (Figure S8).

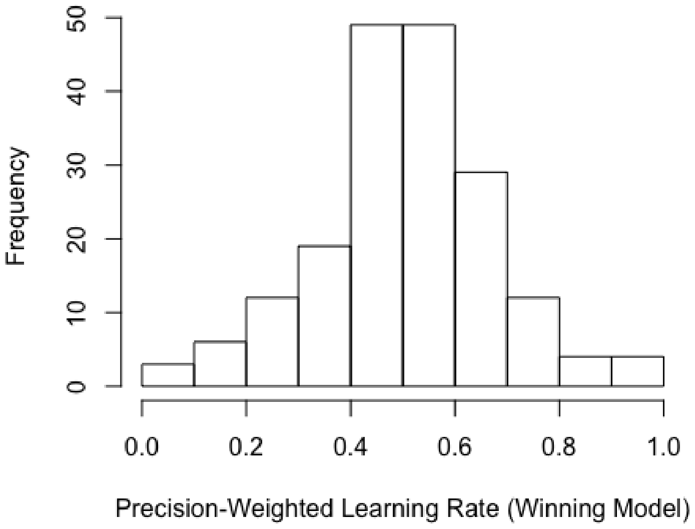

Figure S8: Distribution of the Precision-Weighted Learning Rates as calculated using the winning model's evidence precision proxy (EDI-3-ID).

Model Predictions Validity:

A side-by-side comparison of the model-Predicted vs Actual Prospective Self-Efficacy Beliefs shows that the winning model produces posterior beliefs that overall approximate very well the distribution of the actual posterior beliefs (Figure S9).

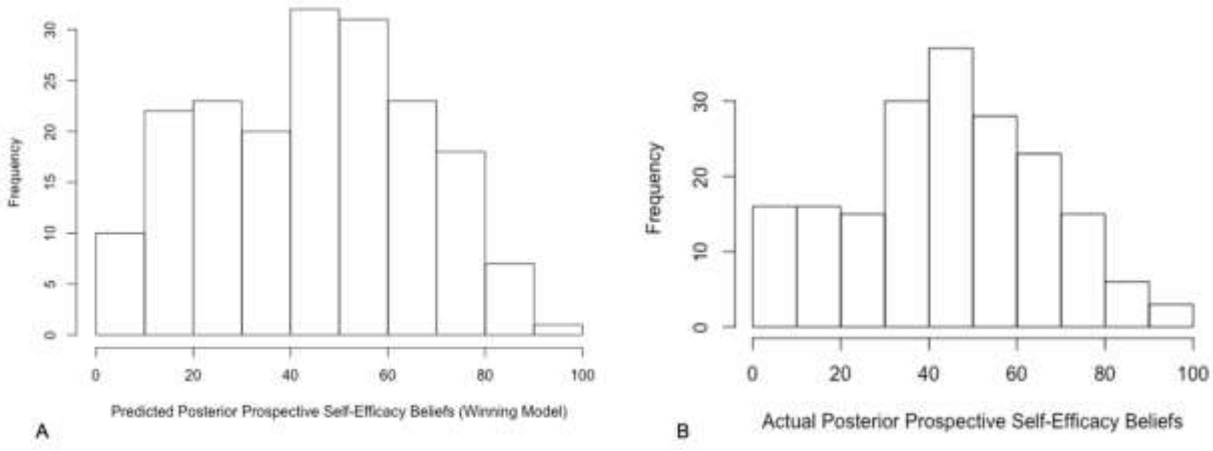

Figure S9: Panel A shows the distribution of the Predicted Posterior Prospective Self-Efficacy Beliefs (based on the winning model). Panel B shows the distribution of participants' actual Posterior Prospective Self-Efficacy Beliefs.

1.4 Exploratory Analyses in the acute and post-acute AN groups

In this part of the Supplementary Material, Results Section, we present the results from the exploratory analyses on counterfactual beliefs, and correlations between our main measures and aspects of insight into illness.

Exploratory Analyses with Everyday, Counterfactual Beliefs

Next, we explored how everyday, counterfactual beliefs on hopes and fears as measured via items developed by the present study's authors (see Table S9) predicted the Prior and Posterior Prospective Self-Efficacy Beliefs of the AN and p-AN groups, separately. We found a significant effect of the Fear subscale on the Posterior Prospective Self-Efficacy Beliefs in both the AN and p-AN groups (Table S21d). Specifically, patients with less overall fear on gaining weight and losing control over their eating behaviour had higher Posterior Prospective Self-Efficacy beliefs. However, we found no significant effects of the fear subscale on patients' Prior Prospective Self-Efficacy Beliefs (Table S21c), or of the hope scale on either their Prior or Prospective Self-Efficacy Beliefs (Table S21a, b). In both the AN and p-AN groups we found a statistical trend effect of overall Counterfactual Belief Scores (i.e., Hope and Fear subscales combined) on patients' Posterior Prospective Self-Efficacy Beliefs (Table S21f), but not on their priors (Table S21e). That is, a higher total Counterfactual Belief Score was indicative of more optimistic Posterior Prospective Self-Efficacy Beliefs. These results yield further validity to our experimental task revealing that our key measure, namely posterior prospective beliefs about interoception following a HCT relates to everyday explicit beliefs patients have about the future.

Table S21. *Exploratory Analyses for Counterfactual Beliefs (Experiment 2)*

S21a: "Hopes" Predicting Prior Prospective Self-Efficacy				
Predictors	β(*SE*)	*t*	*95% CI*	*p*
AN	28.25(*16.03*)	*1.76*	-4.40 – 60.90	*0.088*
Hopes	1.51(*2.26*)	*0.70*	-3.09 – 6.11	*0.508*
p-AN	46.43(*8.36*)	*5.55*	29.37 – 63.49	*<0.001*
Hopes	0.06(*1.31*)	*0.05*	-2.60 – 2.73	*0.962*
S21b: "Fears" Predicting Prior Prospective Self-Efficacy				
AN	32.43(*5.80*)	*5.94*	20.62 – 44.24	*<0.001*
Fears	2.16(*1.47*)	*1.47*	-0.83 – 5.14	*0.150*
p-AN	41.66(*6.56*)	*6.34*	28.27 – 55.04	*<0.001*

Hopes	1.21(*1.23*)	*0.99*	-1.28 – 3.71	*0.329*
S21c: "Hopes" Predicting Posterior Prospective Self-Efficacy				
AN	29.09(*14.08*)	*2.07*	0.41 – 57.77	*0.047*
Hopes	1.38(*1.98*)	*0.70*	-2.66 – 5.41	*0.492*
p-AN	43.54(*10.16*)	*4.28*	22.81 – 64.27	*<0.001*
Hopes	-00.13(*1.59*)	*-0.80*	-3.37 – 3.12	*0.937*
S21d: "Fears" Predicting Posterior Prospective Self-Efficacy				
AN	31.34(*4.96*)	*6.32*	21.23 – 41.44	*<0.001*
Fears	2.52(*1.25*)	*2.01*	-0.04 – 5.07	***0.053***
p-AN	30.71(*7.62*)	*4.03*	15.17 – 46.24	*<0.001*
Fears	2.87(*1.42*)	*2.02*	-0.03 – 5.77	***0.052***
S21e: Overall Counterfactual Beliefs (Hopes and Fears Combined) Predicting Prior Prospective Self-Efficacy				
AN	22.99(*11.47*)	*2.00*	-0.38 – 46.36	*0.054*
Hopes and Fears Combined	3.23(*2.21*)	*1.45*	-1.29 – 7.71	*0.156*
p-AN	35.07(*12.31*)	*2.85*	9.96 – 60.19	*0.008*
Hopes and Fears Combined	2.39(*2.37*)	*1.01*	-2.45 – 7.23	*0.322*
S21f: Overall Counterfactual Beliefs (Hopes and Fears Combined) Predicting Posterior Prospective Self-Efficacy				
AN	21.27(9.89)	2.15	1.13 – 41.41	0.039
Hopes and Fears Combined	3.55(1.90)	1.86	-0.33 – 7.43	0.071
p-AN	17.46(14.42)	1.21	-11.96 – 46.87	0.235
Hopes and Fears Combined	5.18(2.78)	1.86	-0.49 – 10.85	0.072
N.B. Bolded values denote statistical significance (*p<0.05*). Please note that the *p* values here are marginally significant and should be interpreted with caution.				

Additional analyses with items from the EDI-3 questionnaire

Given the similarity between some of the counterfactual belief items from the questionnaire developed by the present study's authors and items from the EDI-3 (Garner, 2004), a validated questionnaire widely used in eating disorders questionnaires, we ran correlational analyses between items of the EDI-3 and Posterior Prospective Self-Efficacy Beliefs in each clinical group separately. Items DT_16, DT_49, B_64, and ID_44 (from the EDI-3; Table S22) are similar to items (9), (11) from the insight questionnaire developed by this study's authors (Table S9). Thus, we wanted to explore whether the pattern of the results would be the same to the results reported in Table S21c when running correlations between the self-efficacy estimates and selected items from the validated, EDI-3 questionnaire. Specifically, we used item 16 ("*I am terrified of gaining weight*"); item 49 ("*If I gain a pound, I worry that*

I will keep gaining"); item 44 ("*I worry that my feelings will get out of control*"); and item 64 ("*When I am upset, I worry that I will start eating*"). However, none of the correlations were statistically significant.

Table S22. *Correlations between Self-Efficacy Beliefs and EDI-3 Items*

	POST PROSP AN	POST PROSP p-AN
DT_16: "I am terrified of gaining weight"	-0.04	0.02
DT_49: "If I gain a pound, I worry that I will keep gaining"	0	-0.03
B_64: "When I am upset, I worry that I will start eating"	0.16	-0.07
ID_44: "I worry that my feelings will get out of control"	-0.11	0.1

N.B. Heatmap with output from correlational analyses. The table presents the *r* values from the correlations. * = *p<.05*, ** = *p<.001*. POST PROSP refers to Posterior Prospective Self-Efficacy Beliefs; AN refers to the Anorexia Nervosa group; p-AN refers to the post-acute Anorexia Nervosa group.

Correlational Analyses with Explicit Beliefs

Insight deficits have been noted in individuals with AN, and we wanted to explore whether there would be a correlation between scores of a questionnaire assessing insight into illness and beliefs about interoception and related measures (here, Prior and Posterior Prospective Self-Efficacy Beliefs, Performance and Learning Rate). None of our correlations between self-efficacy beliefs, performance and learning rates and standalone item and subscale error scores from the insight into illness questionnaire were significant (Table S23).

Table S23. *Correlations with Error Scores*

	PRIORS	POST PROSP	PERFORMANCE	LEARNING RATE 1	LEARNING RATE 2
Item 1	-0.06	0.21	-0.04	0.16	-0.08
Item 2	-0.17	-0.2	0.15	-0.11	0.17
Item 3	-0.14	0.25	0.07	0.17	-0.04
Item 4	-0.15	-0.08	0.1	0.01	0.1
Item 5	0.06	0.05	0.13	0.04	-0.07
Item 6	-0.12	0.01	0.09	-0.08	0.04
Item 7	-0.15	0.19	-0.11	0.14	-0.01
Item 8	-0.08	0.04	-0.07	0.01	-0.06
General, Allocentric Perspective of Self	-0.16	-0.08	0.11	0	0.1
Health Consequences	-0.04	0.03	0.11	-0.03	-0.01
Perspective Taking	-0.12	0.12	-0.1	0.08	-0.03

N.B. Heatmap with results from correlational analyses. The table presents the *r* values from the Pearson correlations. * = *p<.05*, ** = *p<.001*. Items 1-8 refer to the error scores calculated from the respective items of the Insight into Illness Questionnaire (Table S9). The last three rows represent the correlations

with each subscale. PRIORS refers to the Prior Prospective Self-Efficacy Beliefs; POST PROSP refers to the Posterior Prospective Self-Efficacy Beliefs; Learning Rate 1 refers to the precision-weighted learning rate with Performance Confidence as a proxy for precision of the evidence; Learning Rate 2 refers to the precision-weighted learning rate with EDI-3-ID as a proxy for precision of the evidence.

References

American Psychiatric Association (Ed.). (2013). *Diagnostic and statistical manual of mental disorders: DSM-5* (5th ed). American Psychiatric Association.

Arbel, R., Koren, D., Klein, E., & Latzer, Y. (2013). The neurocognitive basis of insight into illness in anorexia nervosa: A pilot metacognitive study. *Psychiatry Research, 209*(3), 604–610. https://doi.org/10.1016/j.psychres.2013.01.009

Baron, R. M., & Kenny, D. A. (1986). The moderator–mediator variable distinction in social psychological research: Conceptual, strategic, and statistical considerations. *Journal of Personality and Social Psychology*, *51*(6), 1173–1182. https://doi.org/10.1037/0022-3514.51.6.1173

Bagby, R. M., Parker, J. D. A., & Taylor, G. J. (1994). The twenty-item Toronto Alexithymia scale—I. Item selection and cross-validation of the factor structure. *Journal of Psychosomatic Research, 38*(1), 23–32. https://doi.org/10.1016/0022-3999(94)90005-1

Beck AT, Steer RA. Manual for the beck anxiety inventory, Psychological Corporation, San Antonio, TX, 1990

Brener, J., & Ring, C. (2016). Towards a psychophysics of interoceptive processes: The measurement of heartbeat detection. *Philosophical Transactions of the Royal Society B: Biological Sciences, 371*(1708), 20160015. https://doi.org/10.1098/rstb.2016.0015

Carleton, R. N., Norton, M. A. P. J., & Asmundson, G. J. G. (2007). Fearing the unknown: A short version of the Intolerance of Uncertainty Scale. *Journal of Anxiety Disorders, 21*(1), 105–117. https://doi.org/10.1016/j.janxdis.2006.03.014

Foa, E. B., Huppert, J. D., Leiberg, S., Langner, R., Kichic, R., Hajcak, G., & Salkovskis, P. M. (2002). The Obsessive-Compulsive Inventory: Development and validation of a short version. *Psychological Assessment*, *14*(4), 485–496. https://doi.org/10.1037/1040-3590.14.4.485

Franz, M., Popp, K., Schaefer, R., Sitte, W., Schneider, C., Hardt, J., Decker, O., & Braehler, E. (2008). Alexithymia in the German general population. *Social Psychiatry and Psychiatric Epidemiology*, *43*(1), 54–62. https://doi.org/10.1007/s00127-007-0265-1

Garfinkel, S. N., Tiley, C., O'Keeffe, S., Harrison, N. A., Seth, A. K., & Critchley, H. D. (2016). Discrepancies between dimensions of interoception in autism: Implications for emotion and anxiety. *Biological Psychology*, *114*, 117–126. https://doi.org/10.1016/j.biopsycho.2015.12.003

Garner, D. M. (2004). *Eating Disorder Inventory—3 (EDI-3). Professional Manual.* Odessa, FL: Psychological Assessment Resources, 1.

Gloster, A. T., Rhoades, H. M., Novy, D., Klotsche, J., Senior, A., Kunik, M., Wilson, N., & Stanley, M. A. (2008). Psychometric properties of the Depression Anxiety and Stress Scale-21 in older primary care patients. *Journal of Affective Disorders*, *110*(3), 248–259. https://doi.org/10.1016/j.jad.2008.01.023

González-Arias, M., Martínez-Molina, A., Galdames, S., & Urzúa, A. (2018). Psychometric Properties of the 20-Item Toronto Alexithymia Scale in the Chilean Population. *Frontiers in Psychology*, *9*, 963. https://doi.org/10.3389/fpsyg.2018.00963

Grant, D. A., & Berg, E. (1948). A behavioral analysis of degree of reinforcement and ease of shifting to new responses in a Weigl-type card-sorting problem. *Journal of Experimental Psychology*, *38*(4), 404–411. https://doi.org/10.1037/h0059831

Hamilton M, A rating scale for depression, J. Neurol. Neurosurg. Psychiatry 23 (1960) 56–62.

Hamilton M, The assessment of anxiety states by rating, Br. J. Med. Psychol. 32 (1) (1959) 50–55.

Honkalampi, K., Hintikka, J., Laukkanen, E., & Viinamäki, J. L. H. (2001). Alexithymia and Depression: A Prospective Study of Patients With Major Depressive Disorder. *Psychosomatics*, *42*(3), 229–234. https://doi.org/10.1176/appi.psy.42.3.229

Kinnaird, E., Stewart, C., & Tchanturia, K. (2020). Interoception in Anorexia Nervosa: Exploring Associations With Alexithymia and Autistic Traits. *Frontiers in Psychiatry*, *11*. https://www.frontiersin.org/articles/10.3389/fpsyt.2020.00064

Knapp-Kline, K., & Kline, J. P. (2005). Heart rate, heart rate variability, and heartbeat detection with the method of constant stimuli: Slow and steady wins the race. *Biological Psychology*, *69*(3), 387–396. https://doi.org/10.1016/j.biopsycho.2004.09.002

Konstantakopoulos, G., Tchanturia, K., Surguladze, S. A., & David, A. S. (2011). Insight in eating disorders: Clinical and cognitive correlates. *Psychological Medicine*, *41*(9), 1951–1961. https://doi.org/10.1017/S0033291710002539

Konstantakopoulos, G., Georgantopoulos, G., Gonidakis, F., Michopoulos, I., Stefanatou, P., & David, A. S. (2020). Development and validation of the schedule for the assessment of insight in eating disorders (SAI-ED). *Psychiatry Research*, *292*, 113308. https://doi.org/10.1016/j.psychres.2020.113308

Lovibond, P. F., & Lovibond, S. H. (1995). The structure of negative emotional states: Comparison of the Depression Anxiety Stress Scales (DASS) with the Beck Depression and Anxiety Inventories. *Behaviour Research and Therapy*, *33*(3), 335–343. https://doi.org/10.1016/0005-7967(94)00075-U

Meyer, T. J., Miller, M. L., Metzger, R. L., & Borkovec, T. D. (1990). Development and validation of the penn state worry questionnaire. *Behaviour Research and Therapy*, *28*(6), 487–495. https://doi.org/10.1016/0005-7967(90)90135-6

Richard, A., Meule, A., Georgii, C., Voderholzer, U., Cuntz, U., Wilhelm, F. H., & Blechert, J. (2019). Associations between interoceptive sensitivity, intuitive eating, and body mass index in patients with anorexia nervosa and normal-weight controls. *European Eating Disorders Review*, *27*(5), 571–577. https://doi.org/10.1002/erv.2676

Sapra, A., Malik, A., & Bhandari, P. (2023). Vital Sign Assessment. In *StatPearls*. StatPearls Publishing. http://www.ncbi.nlm.nih.gov/books/NBK553213/

Saramandi, A., Crucianelli, L., Koukoutsakis, A., Nisticò, V., Baiza, A., Goeta, D., Demartini, B., Gambini, O., Jenkinson, P., & Fotopoulou, A. (2022). *Belief updating about Interoception and Body Size Estimation in Anorexia Nervosa*. PsyArXiv. https://doi.org/10.31234/osf.io/rntsf

Shields, S. A., Mallory, M. E., & Simon, A. (1989). The Body Awareness Questionnaire: Reliability and Validity. *Journal of Personality Assessment*, *53*(4), 802–815. https://doi.org/10.1207/s15327752jpa5304_16

Spielberger, C. D., Gonzalez-Reigosa, F., Martinez-Urrutia, A., Natalicio, L. F. S., & Natalicio, D. S. (1971). *The State-Trait Anxiety Inventory*. *5*. https://journal.sipsych.org/index.php/IJP/article/view/620

Unal, A., Altug, F., Erden, A., Cavlak, U., & Senol, H. (2021). Validity and reliability of the Body Awareness Questionnaire in patients with non-specific chronic low back pain. *Acta Neurologica Belgica*, *121*(3), 701–705. https://doi.org/10.1007/s13760-020-01399-y

Van der Ploeg, H. M. (1980). Validity of the Zelf-Beoordelings-Vragenlijst (A Dutch version of the Spielberger State-Trait Anxiety Inventory). *Nederlands Tijdschrift Voor de Psychologie En Haar Grensgebieden*, *35*, 243–249.

Van Rijsoort, S., Emmelkamp, P., & Vervaeke, G. (1999). The Penn State Worry Questionnaire and the Worry Domains Questionnaire: Structure, reliability and validity. *Clinical Psychology & Psychotherapy*, *6*(4), 297–307. https://doi.org/10.1002/(SICI)1099-0879(199910)6:4<297::AID-CPP206>3.0.CO;2-E

Wickham, H. (2016). Data Analysis. In H. Wickham (Ed.), *Ggplot2: Elegant Graphics for Data Analysis* (pp. 189–201). Springer International Publishing. https://doi.org/10.1007/978-3-319-24277-4_9

Wootton, B. M., Diefenbach, G. J., Bragdon, L. B., Steketee, G., Frost, R. O., & Tolin, D. F. (2015). A contemporary psychometric evaluation of the Obsessive Compulsive Inventory—Revised (OCI-R). *Psychological assessment*, *27*(3), 874
